# Supplementary material for: Focused ultrasound-mediated lipid nanoparticle delivery for brain gene editing
Source: Mol Ther Nucleic Acids. 2026 Jul 14;37(3):103012. doi: 10.1016/j.omtn.2026.103012 (PMC13453546; doi:10.1016/j.omtn.2026.103012)
Supplement: Supplementary file 1 [file mmc1.pdf]

OMTN, Volume *37*

Supplemental information

Focused ultrasound-mediated lipid nanoparticle delivery for brain gene editing

Kaiyuan Zheng, Fotios N. Tsitsos, Elisa E. Konofagou, and Kam W. Leong

SUPPLEMENTAL FIGURES

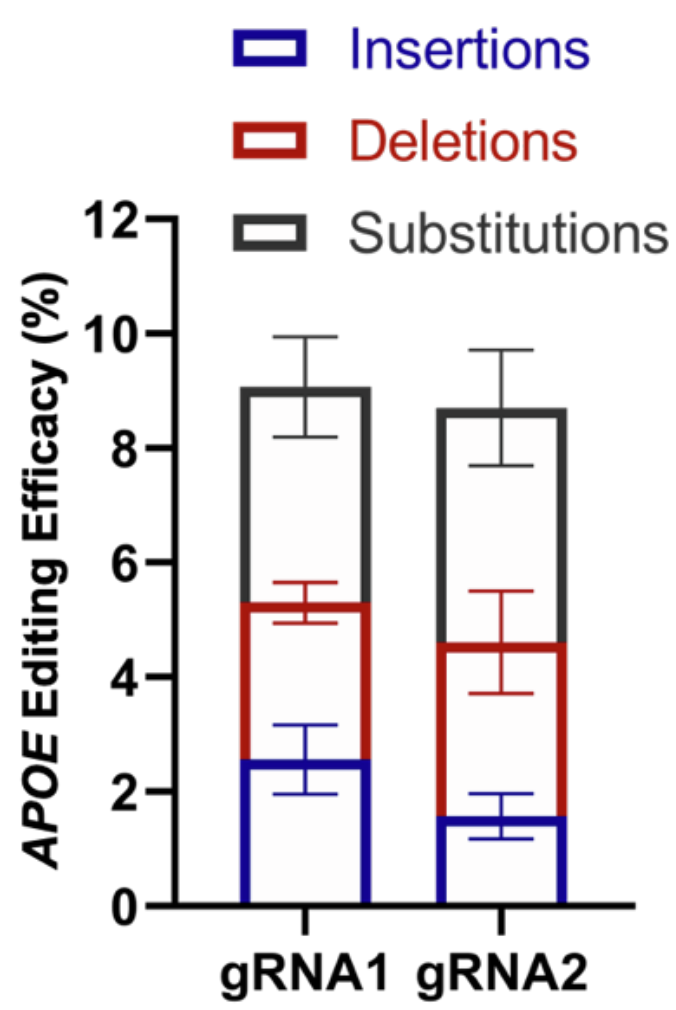

Figure S1. *APOE4* editing profiles at two gRNA target sites. Stacked bars show the percentage of insertions, deletions, and substitutions contributing to total editing efficacy at each target site.

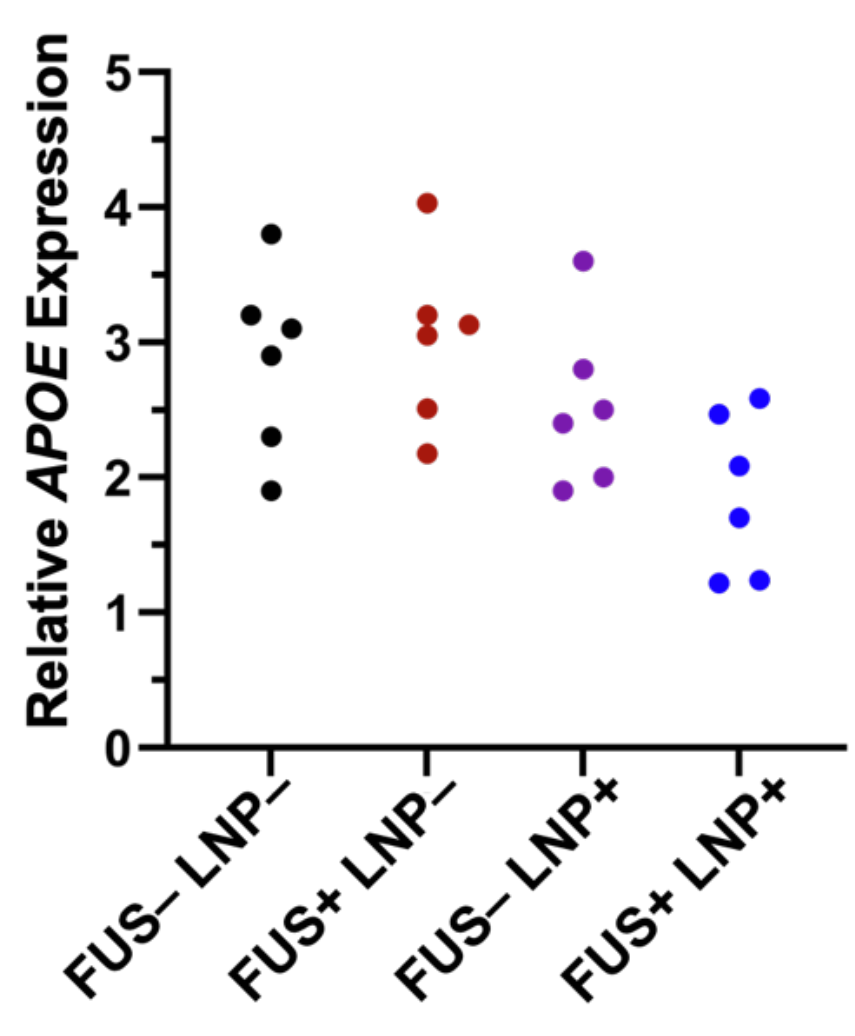

Figure S2. Raw values used to quantify relative *APOE* mRNA expression levels in the hippocampus by RT-qPCR.

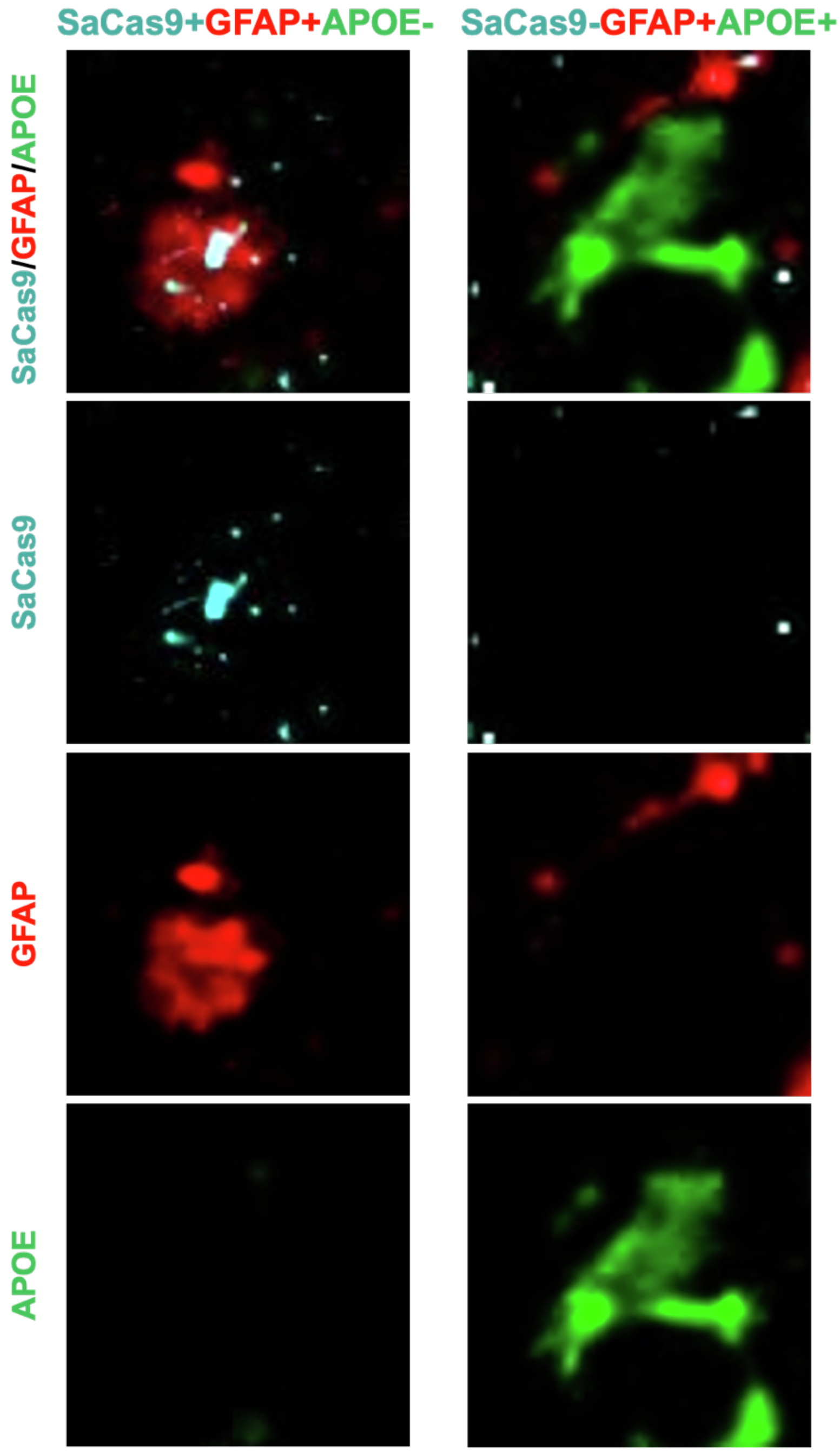

Figure S3. Enlarged representative confocal images showing *SaCas9*$^{+}$*GFAP*$^{+}$*APOE*$^{-}$ cell and *SaCas9*$^{-}$*GFAP*$^{+}$*APOE*$^{+}$ cell detected by RNA ISH.

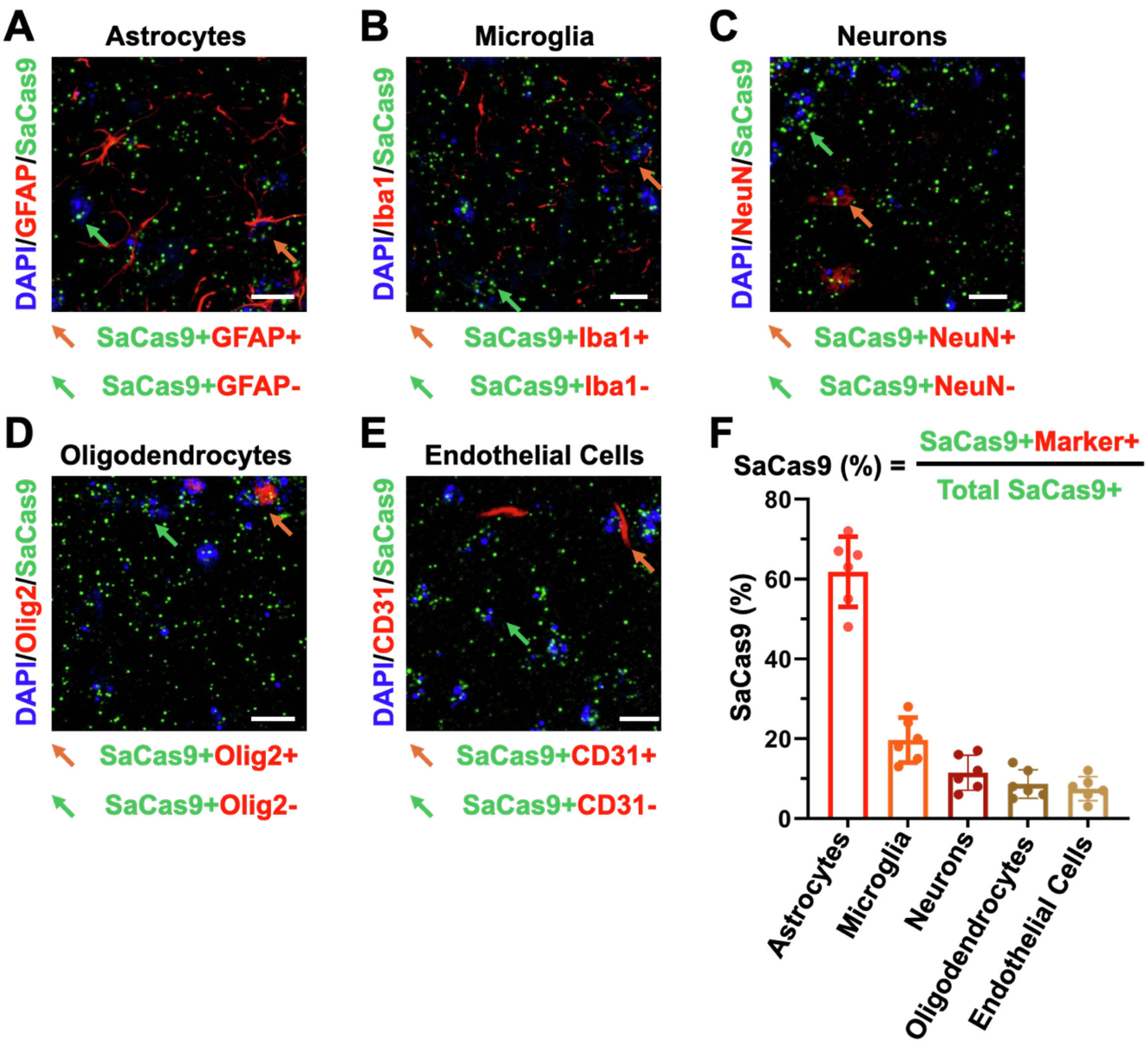

Figure S4. Representative confocal images showing *SaCas9* mRNA (green) distribution in hippocampal astrocytes stained for GFAP (A, red), microglia stained for Iba1 (B, red), neurons stained for NeuN (C, red), oligodendrocytes stained for Olig2 (D, red), and endothelial cells stained for CD31 (E, red). Scale bars, 20 µm. (F) Quantification of *SaCas9* mRNA distribution across different cell types.

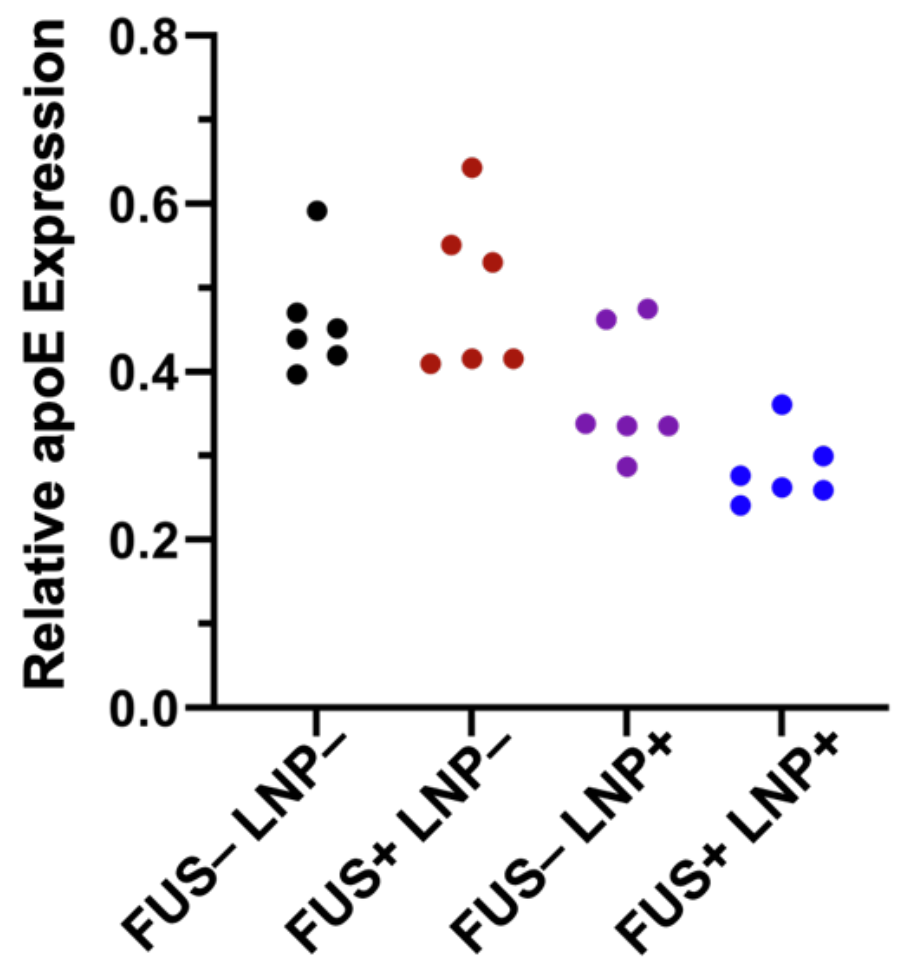

Figure S5. Raw values used to quantify relative apoE4 protein expression levels in the hippocampus by ELISA.

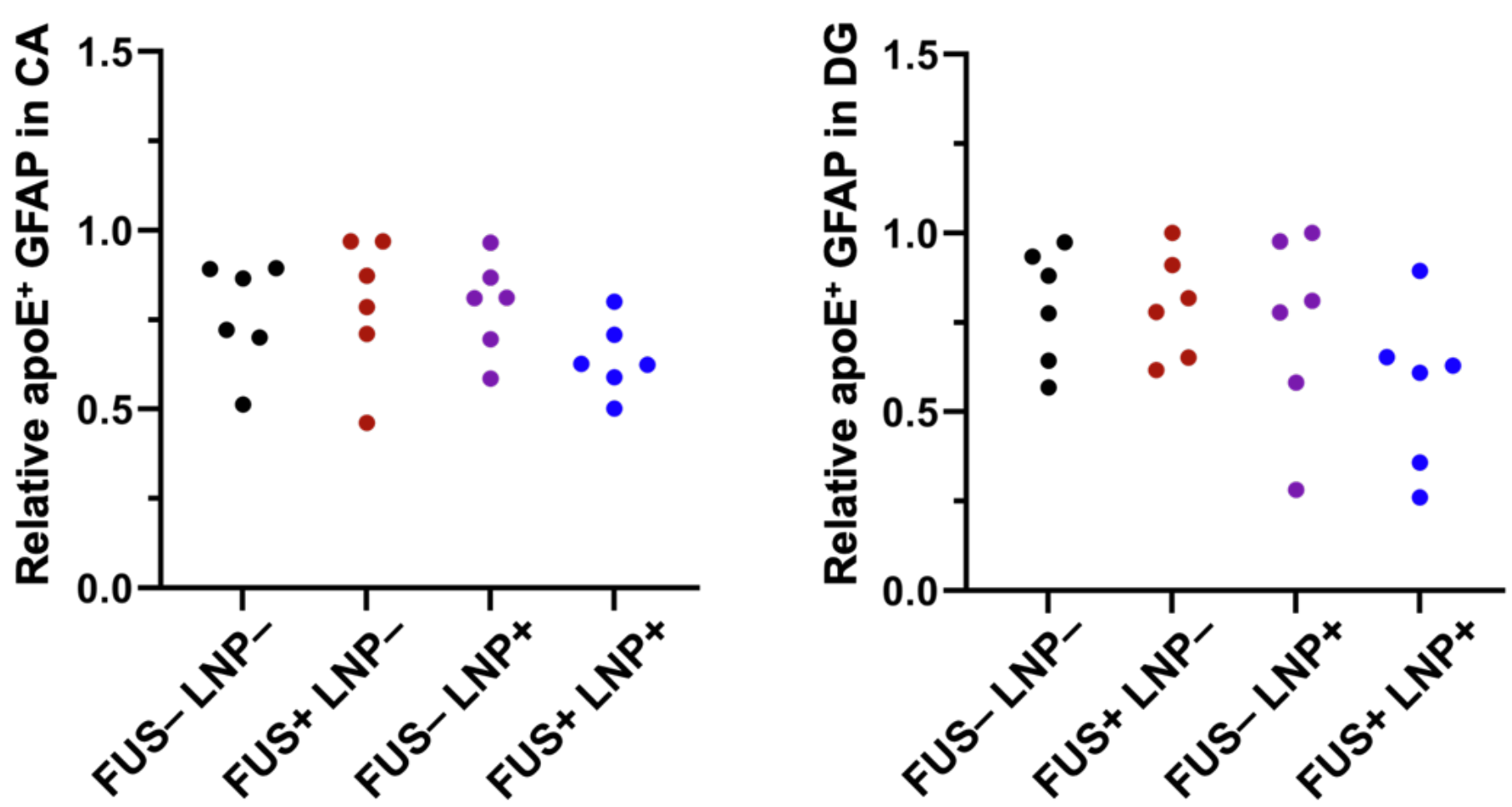

Figure S6. Raw values used to quantify apoE4-positive GFAP-staining astrocytes in the CA and DG regions.

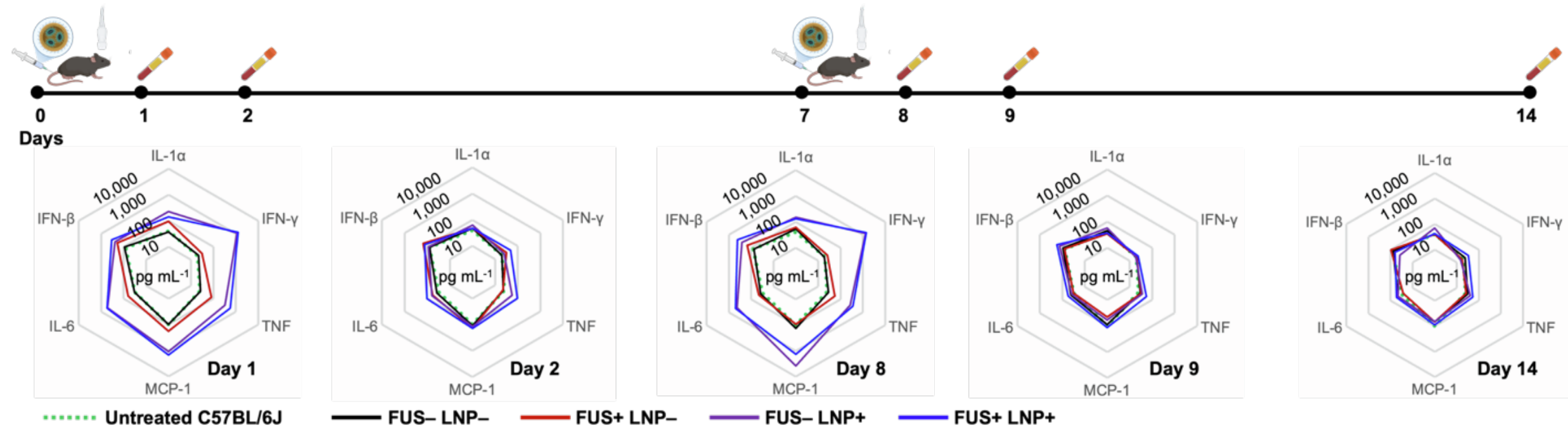

Figure S7. Plasma pro-inflammatory cytokine levels following treatment, with experimental timeline shown in the top panel (created with BioRender). "FUS-LNP-" indicates APOE4KI mice without any treatment, "FUS+LNP-" APOE4KI mice received FUS alone without i.v. LNPs, "FUS-LNP+" APOE4KI mice received i.v. LNPs alone without FUS, and "FUS+LNP+" APOE4KI mice received both FUS and i.v. LNPs. "Untreated C57BL/6J" denotes wild-type C57BL/6J mice without any treatment.

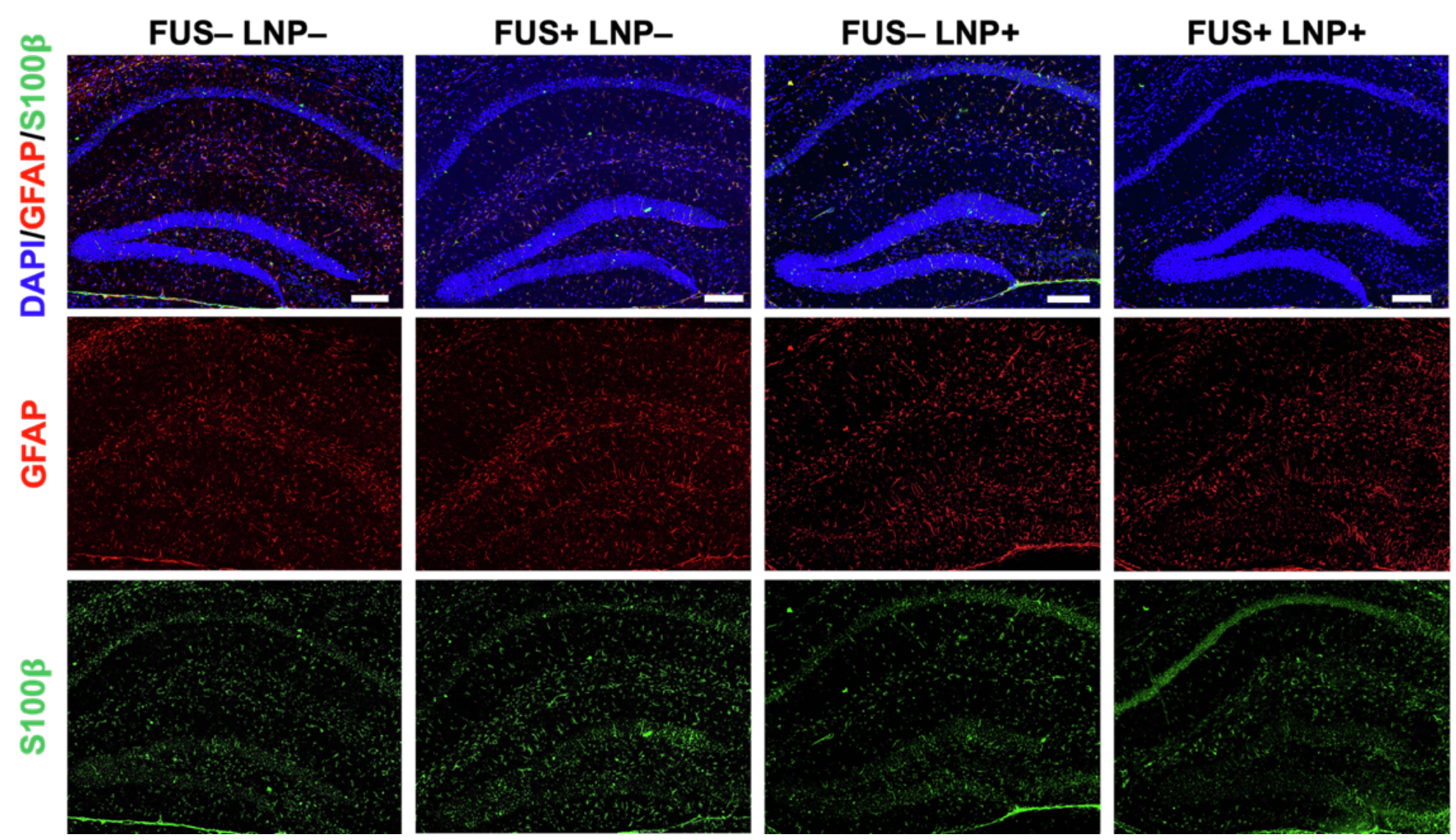

Figure S8. Representative confocal images of IF staining for GFAP (red), S100β (green), and DAPI (blue) in the hippocampus. Scale bars, 200 μm.

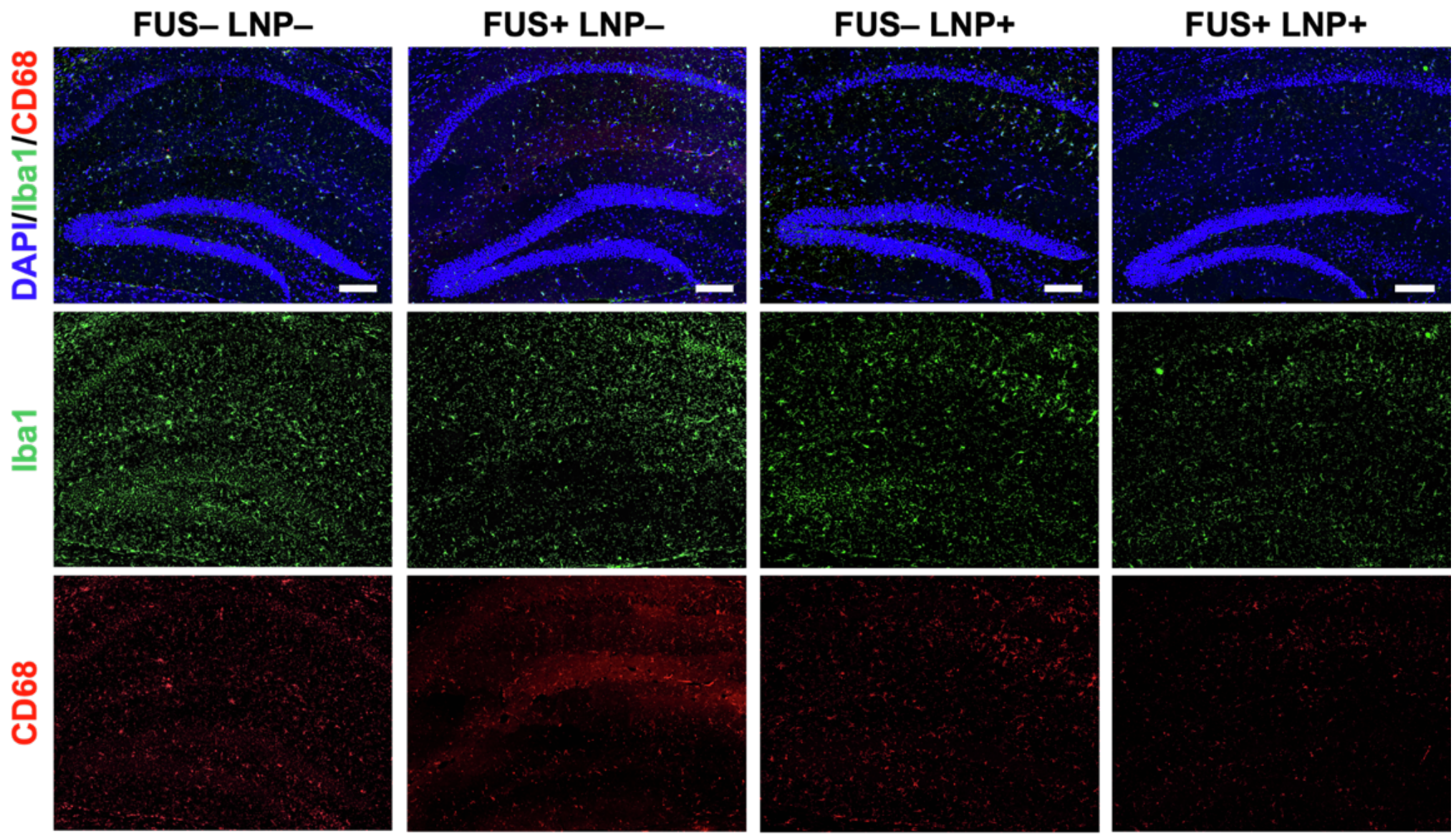

Figure S9. Representative confocal images of IF staining for Iba1 (green), CD68 (red), and DAPI (blue) in the hippocampus. Scale bars, 200 μm.

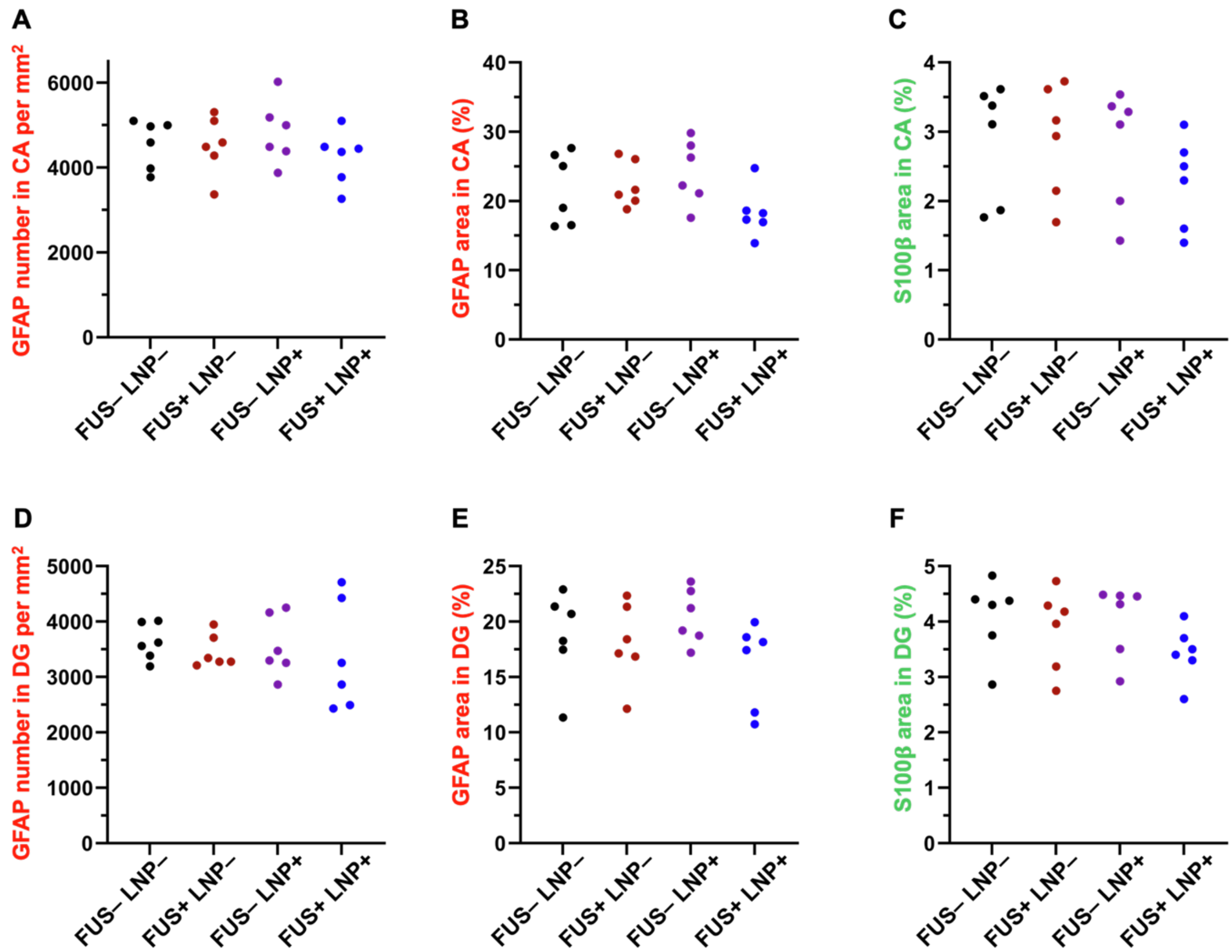

Figure S10. Raw values used to quantify astrocyte markers in the CA and DG regions. Raw values for the number of GFAP (A), coverage area of GFAP (B), and coverage area of S100β (C) in the CA region, and the number of GFAP (D), coverage area of GFAP (E), and coverage area of S100β (F) in the DG region.

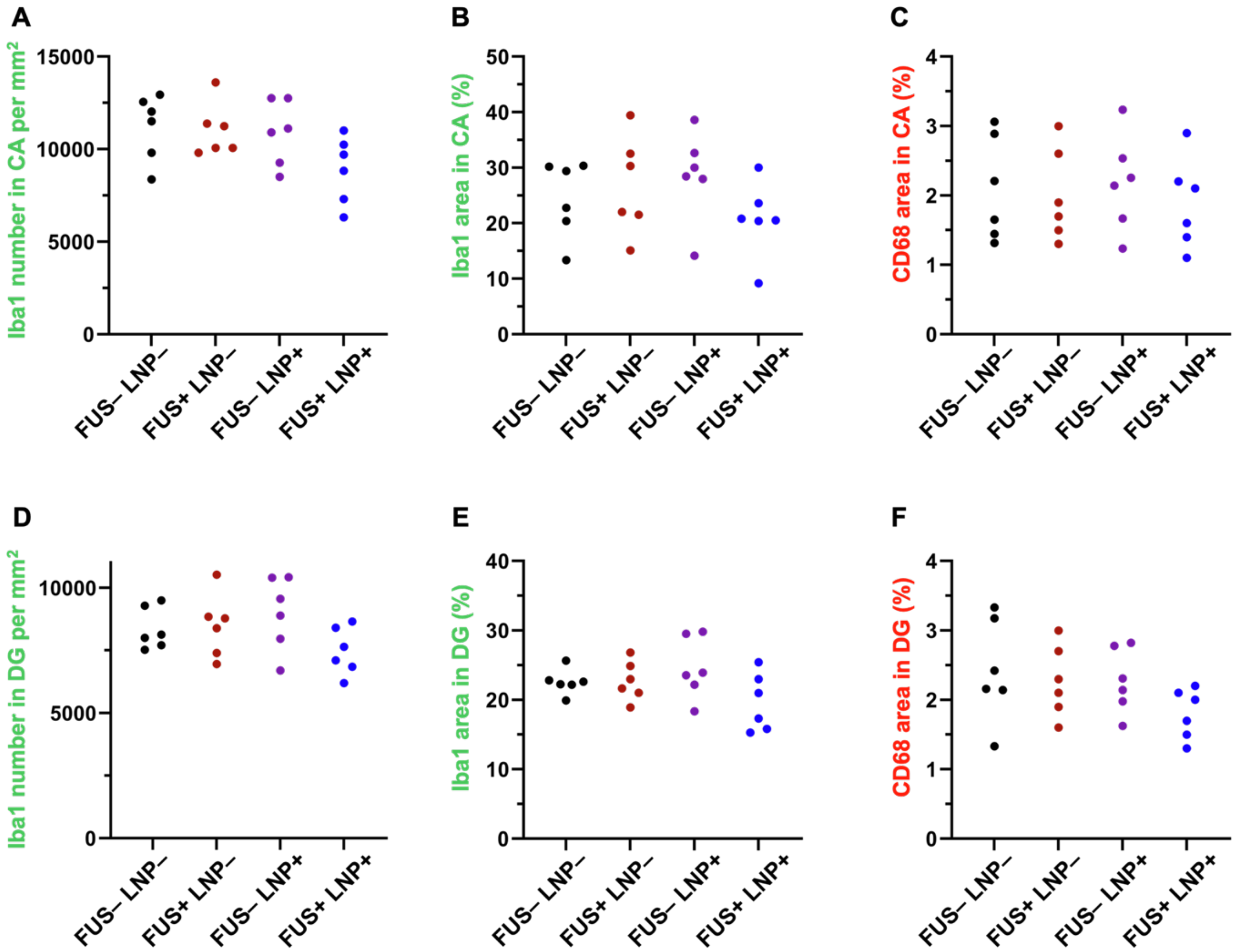

Figure S11. Raw values used to quantify microglia markers in the CA and DG regions. Raw values for the number of Iba1 (A), coverage area of Iba1 (B), and coverage area of CD68 (C) in the CA region, and the number of Iba1 (D), coverage area of Iba1 (E), and coverage area of CD68 (F) in the DG region.

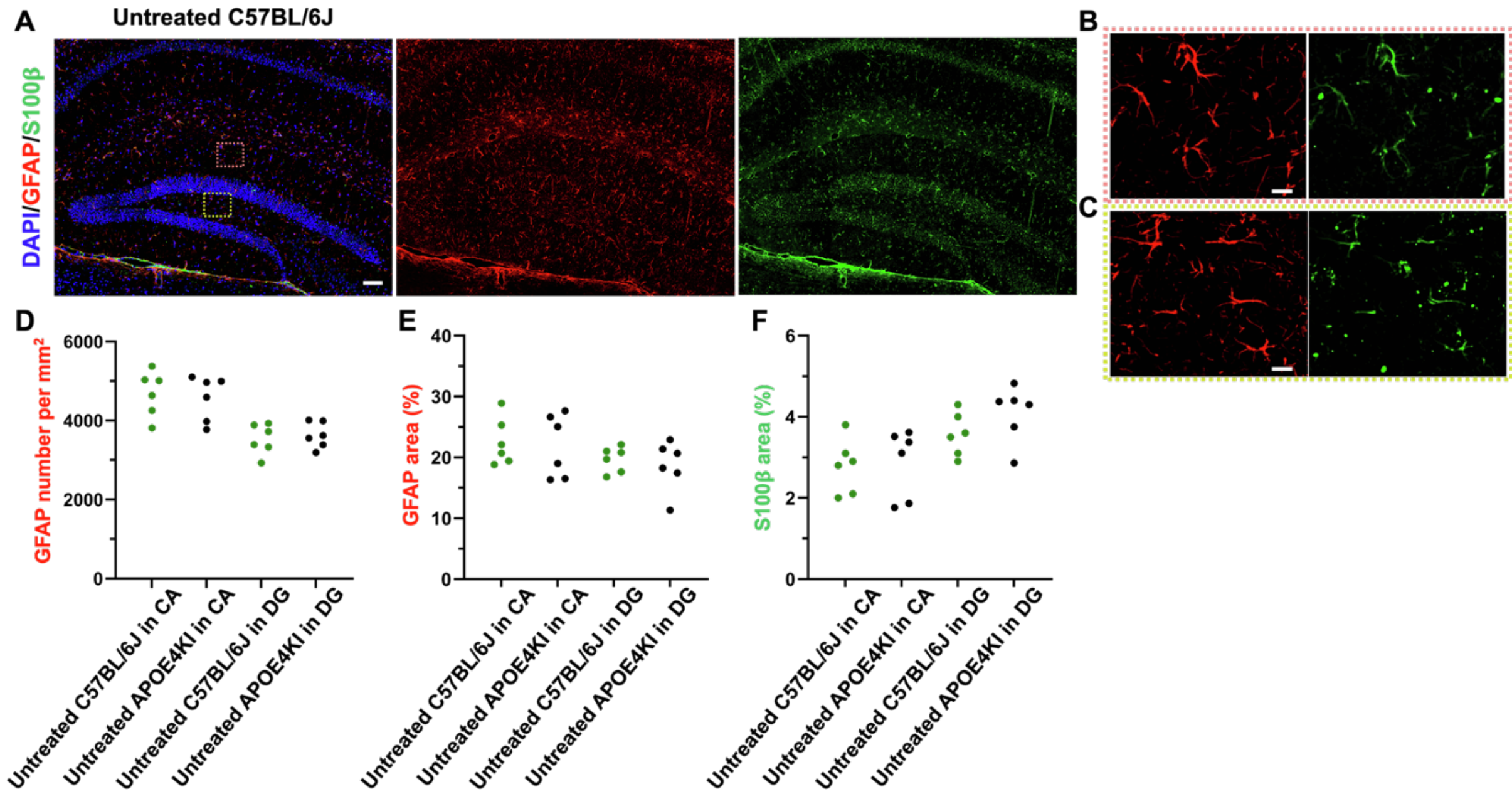

Figure S12. IF staining of astrocyte markers in untreated wild-type C57BL/6J mice. (A-C) IF staining of untreated wild-type C57BL/6J mice for GFAP (red), S100β (green), and DAPI (blue). (A) Representative confocal images in the hippocampus. Scale bars, 200 μm. Pink and yellow boxes indicate ROIs in the CA (B) and DG (C) regions, respectively. Scale bars, 20 μm. (D-F) Raw values for number of GFAP (D), coverage area of GFAP (E), and coverage area of S100β (F) in the CA and DG regions of untreated wild-type C57BL/6J mice and untreated APOE4KI mice (FUS-LNP-).

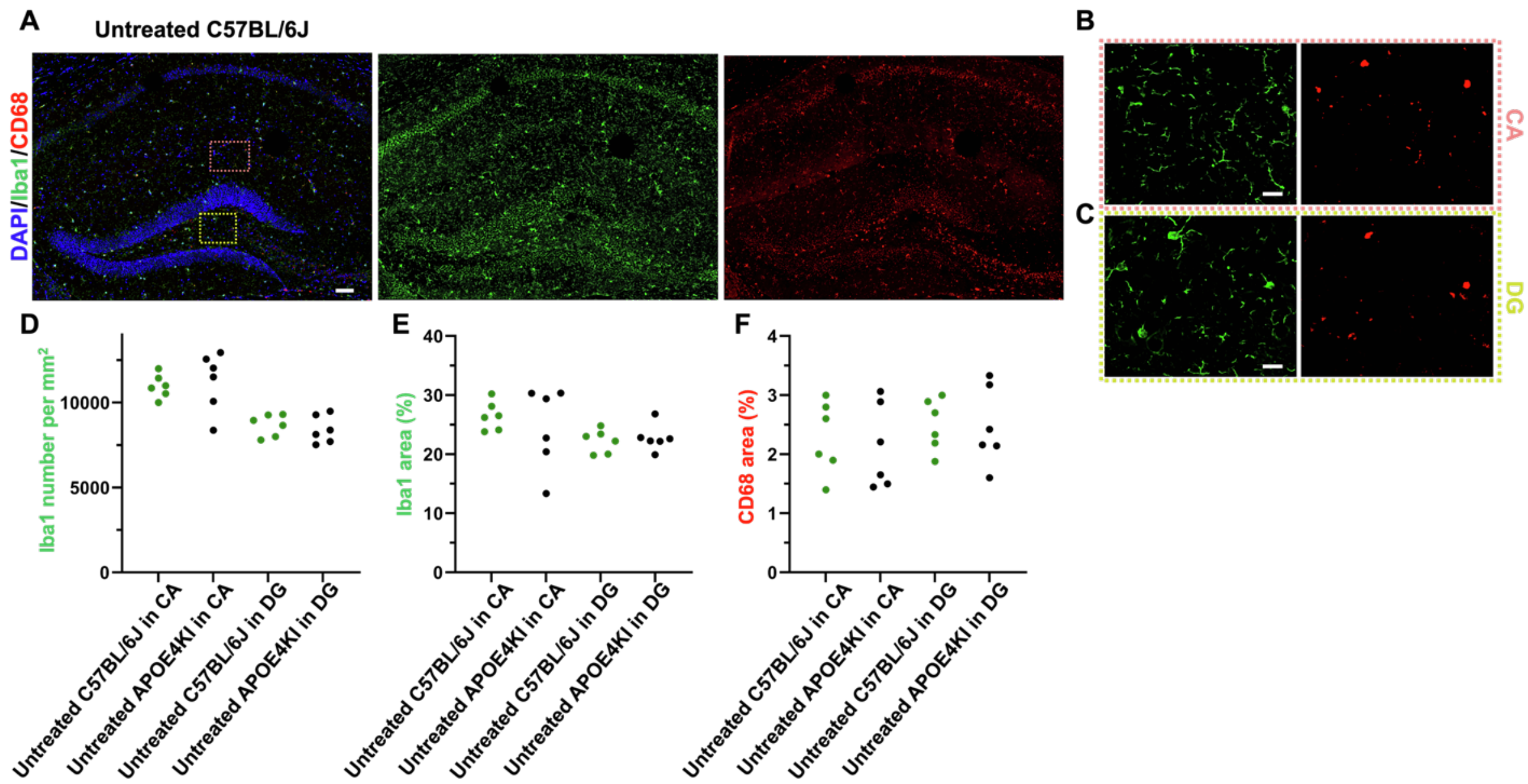

Figure S13. IF staining of microglia markers in untreated wild-type C57BL/6J mice. (A-C) IF staining of untreated wild-type C57BL/6J mice for Iba1 (green), CD68 (red), and DAPI (blue). (A) Representative confocal images in the hippocampus. Scale bars, 200 µm. Pink and yellow boxes indicate ROIs in the CA (B) and DG (C) regions, respectively. Scale bars, 20 µm. (D-F) Raw values for number of Iba1 (D), coverage area of Iba1 (E), and coverage area of CD68 (F) in the CA and DG regions of untreated wild-type C57BL/6J mice and untreated APOE4KI mice (FUS-LNP-).

SUPPLEMENTAL METHODS

Characterization of pDNA-LNPs

Particle size and zeta potential of pDNA-LNPs were measured using ZetaSizer Nano ZS90 (Malvern Panalytical), and values are reported as mean ± SD (n = 3). Encapsulation efficiency (EE) of pDNA in LNPs was quantified using Quant-iT™ PicoGreen™ dsDNA Assay Kits (Invitrogen). The concentration of unencapsulated pDNA was measured in intact pDNA-LNPs, whereas the concentration of total pDNA was measured after disruption of the LNPs with Triton X-100. EE was calculated according to the following formula:

$$\mathrm{EE}\ (\%) = \frac{\text{total pDNA} - \text{unencapsulated pDNA}}{\text{total pDNA}} \times 100 \qquad [S1]$$

Mice

All animal studies were performed in accordance with protocols AC-AABU2665, AC-AABG4559, and AC-AABX4654 approved by the Columbia University Institutional Animal Care and Use Committee (IACUC). Transgenic APOE4KI mice (JAX Stock No. 027894), which carry homozygous humanized APOE4 alleles [B6(SJL)-Apoe$^{tm1.1(APOE*4)Adiuj}$/J], were purchased from The Jackson Laboratory. The wild-type C57BL/6J mice (JAX Stock No. 000664) were used as untreated controls. All animals were housed with littermates in a temperature-controlled facility under a 12-hour light/dark cycle and were provided ad libitum access to standard chow and water.

In vivo gene editing validation

Genomic DNA and RNA were extracted from the left and right hippocampi using the Quick DNA/RNA Miniprep Plus Kit (Zymo) according to the manufacturer's instructions.

Genomic DNA was amplified using Platinum SuperFi II PCR Master Mix (Invitrogen) with the following cycling conditions: 98 °C for 10 s, 60 °C for 10 s, and 72 °C for 15 s, repeated for 35 cycles. PCR amplicons were purified using the GeneJET Gel Extraction and DNA Cleanup Micro Kit (Thermo Scientific) and quantified with Quant-iT™ PicoGreen™ dsDNA Assay Kits (Invitrogen) according to the manufacturer's protocols. Purified amplicons were sequenced by Plasmidsaurus, and the sequencing data were analyzed using CRISPResso2. Editing efficiency was calculated from amplicon deep sequencing as the percentage of sequencing reads containing insertions, deletions, and substitutions around each predicted cut site.

Extracted RNA was reverse-transcribed into cDNA using the iScript cDNA Synthesis Kit (Bio-Rad) according to the manufacturer's instructions. Reverse transcription-quantitative polymerase chain reaction (RT-qPCR) was performed on QuantStudio 3 Real-Time PCR System (Applied Biosystems) using SsoAdvanced SYBR Green Supermix (Bio-Rad) according to the manufacturer's protocol. APOE4 mRNA expression was normalized to the housekeeping gene 18S rRNA. For each mouse, the relative ratio was calculated by comparing the normalized APOE4 expression level in the left hippocampus (FUS+LNP+) with that in the right hippocampus (FUS-LNP+) to compare between the sonicated and contralateral hippocampus within the same animal, in order to account for inter-animal variability. In the control group, the ratio was calculated by comparing the left hippocampus (FUS+LNP-) with the right hippocampus (FUS-LNP-).

RNA *in situ* hybridization (ISH)

RNA ISH was performed on paraffin-embedded brain sections. Sections were deparaffinized and pretreated using the RNAscope Multiplex Fluorescent Reagent Kit v2 (Advanced Cell Diagnostics, 323100) according to the manufacturer's instructions. Probes targeting Mm-Gfap (313211),

saCas9 (501621-C2), and Hs-APOE (433091-C3) were used in combination with TSA Vivid Fluorophore 520 (323271), TSA Vivid Fluorophore 570 (323272), and TSA Vivid Fluorophore 650 (323273) according to the manufacturer's protocol.

To quantify *SaCas9* mRNA expression across different cell types, including astrocytes, microglia, neurons, oligodendrocytes, and endothelial cells, ISH was combined with immunofluorescence according to the manufacturer's protocol. The following primary antibodies were used: chicken anti-GFAP (1:500, Abcam, ab4674), rabbit anti-Iba1 (1:500, Wako, 019-19741), rabbit anti-NeuN (1:800, Abcam, ab177487), rabbit anti-Olig2 (1:200, Abcam, ab109186), and rabbit anti-CD31 (1:1000, Abcam, ab281583). The following secondary antibodies were used: Alexa Fluor 488 goat anti-rabbit (1:500, Abcam, ab150077) and Alexa Fluor 594 goat anti-chicken (1:500, Abcam, ab150172). As for *SaCas9* mRNA, the saCas9 (501621-C2) probe was used in combination with TSA Vivid Fluorophore 650 (323273).

Imaging was performed using a Nikon Ti2 inverted microscope equipped with an AXR resonant spectral scanning confocal unit at 60× magnification. Cells were manually counted and classified as positive for a target gene if they contained at least five RNA spots within the corresponding cell-marker-defined region.

Enzyme-linked immunosorbent assay (ELISA)

ApoE4 protein levels in brain lysates from the left and right hippocampi were quantified using the Human Apolipoprotein E4 ELISA Kit (Abcam) according to the manufacturer's instructions. ApoE4 expression was normalized to the housekeeping protein GAPDH using the Mouse GAPDH ELISA Kit (AFG Scientific) according to the manufacturer's protocol. For each mouse, the relative ratio was calculated by comparing the normalized apoE4 expression level in the left hippocampus

(FUS+LNP+) with that in the right hippocampus (FUS-LNP+). In the control group, the ratio was calculated by comparing the left hippocampus (FUS+LNP-) with the right hippocampus (FUS-LNP-).

Immunofluorescence (IF) staining

Collected brains were post-fixed in 4% PFA, embedded in paraffin, and coronally sectioned at a thickness of 5 μm. Sections were deparaffinized in xylene, rehydrated through a graded ethanol series, and subjected to antigen retrieval using eBioscience IHC Antigen Retrieval Solution (Invitrogen). Sections were then rinsed in TBS-Tween 20 (TBST, Thermo Scientific) and blocked with 10% goat serum in TBST for 1 h at room temperature. Primary antibodies diluted in blocking buffer were applied to the sections, which were then incubated overnight at 4 °C. The following primary antibodies were used: rabbit anti-ApoE (1:500, Abcam, ab52607), chicken anti-GFAP (1:500, Abcam, ab4674), rabbit anti-S100β (1:500, Abcam, ab52642), rabbit anti-Iba1 (1:500, Wako, 019-19741), and chicken anti-CD68 (1:500, Abcam, ab318303). After three 10-min washes in TBST, sections were incubated with secondary antibodies diluted in blocking buffer for 2 h at room temperature. The following secondary antibodies were used: Alexa Fluor 488 goat anti-rabbit (1:500, Abcam, ab150077) and Alexa Fluor 594 goat anti-chicken (1:500, Abcam, ab150172). Finally, sections were washed three times for 10 min each in TBST and mounted with ProLong Gold Antifade Mountant with DAPI (Invitrogen).

Imaging was performed using a Nikon Ti2 inverted microscope equipped with an AXR resonant spectral scanning confocal unit at 60× magnification. Colocalization of ApoE4 and GFAP, as well as the number and coverage area of GFAP-positive and Iba1-positive cells, were quantified using Imaris 10.2 (Oxford Instruments) with the AI-based Filament Tracer for linear branching

networks. The coverage areas of S100β and CD68 were quantified using ImageJ. Quantification was performed separately for the cornu ammonis (CA) and dentate gyrus (DG) regions of the hippocampus, and marker abundance was expressed as cell number per mm^2 or coverage area (%). For each mouse, the relative ratio of each marker in the left hippocampus (FUS+LNP+) to that in the right hippocampus (FUS-LNP+) was calculated. In the control group, the ratio of the left hippocampus (FUS+LNP-) to the right hippocampus (FUS-LNP-) was calculated.

Pro-inflammatory cytokine measurements

Blood was collected from the facial vein using a sterile 5 mm lancet into capillary blood collection tubes pre-coated with lithium heparin at designated time points after treatment. Samples were centrifuged at 2,000 × g for 10 min at 4 °C, and the plasma supernatant was collected and stored at -80 °C until analysis. Plasma cytokine levels were quantified by ELISA according to the manufacturers' instructions using kits from Invitrogen for IFN-β, IL-1α, IFN-γ, TNF-α, MCP-1, and IL-6.

Statistics

Statistical analyses were performed using Prism 9.0 (GraphPad). Data are presented as mean ± standard error of the mean (s.e.m.). Statistical significance was assessed using unpaired two-tailed Student's t-tests or two-way ANOVA followed by Dunnett's multiple comparisons test comparing each group with the FUS-LNP- control group. Differences were considered statistically significant at $*P < 0.05$, $**P < 0.01$, $***P < 0.001$, and $****P < 0.0001$.
