# Supplementary material for: Focused ultrasound-mediated lipid nanoparticle delivery for brain gene editing
Source: Mol Ther Nucleic Acids. 2026 Jul 14;37(3):103012. doi: 10.1016/j.omtn.2026.103012 (PMC13453546; doi:10.1016/j.omtn.2026.103012)
Supplement: Supplementary file 2 [file mmc2.pdf]

Molecular Therapy
Nucleic Acids
Brief report

Focused ultrasound-mediated lipid nanoparticle delivery for brain gene editing

Kaiyuan Zheng,[1] Fotios N. Tsitsos,[1] Elisa E. Konofagou,[1,2] and Kam W. Leong[1,3]

[1]Department of Biomedical Engineering, Columbia University, New York NY 10027, USA; [2]Department of Radiology, Columbia University Medical Center, New York NY 10032, USA; [3]Department of Systems Biology, Columbia University Medical Center, New York NY 10032, USA

Efficient brain gene editing remains constrained by the lack of delivery platforms that combine efficacy, spatial precision, and translational potential. Compared with viral vectors, lipid nanoparticles (LNPs) offer larger cargo capacity and lower immunogenicity for repeat dosing. However, their brain delivery is restricted by the blood-brain barrier (BBB). Here, we show that focused ultrasound (FUS)-mediated BBB opening enables systemic delivery of CRISPR-encoding plasmid DNA (pDNA)-LNPs for brain gene editing. Using a pDNA construct containing astrocyte-targeting GfaABC1D promoter and dual guide RNAs targeting apolipoprotein E4 (*APOE4*), the strongest genetic risk factor for Alzheimer's disease, we achieved efficient *APOE4* knockdown, with reduced *APOE4* mRNA and apoE4 protein expression, and attenuated astrocytes and microglial activation. These results establish FUS-mediated pDNA-LNP delivery as a non-invasive, non-viral strategy for brain gene editing that provides spatial control and cell-type-specific expression, while accommodating large genetic payload and enabling repeatable dosing.

INTRODUCTION

Lipid nanoparticles (LNPs) are versatile non-viral platforms for nucleic acid delivery. Compared with adeno-associated viruses (AAVs), LNPs impose fewer constraints on cargo size and may support repeat dosing due to lower immunogenicity.[1] Although LNPs are best known for RNA delivery, they can also encapsulate plasmid DNA (pDNA), offering several advantages, such as availability of cell-type-specific promoters, prolonged expression, improved stability, and lower cost.[2] However, pDNA-LNPs remain less studied due to lower delivery efficiency and concerns about innate immune activation. Approaches that improve delivery efficiency and tissue specificity are therefore critical. To enable efficient delivery to the brain, one of the most challenging organs to target, focused ultrasound (FUS) provides a non-invasive and spatially controlled technique for transient blood-brain barrier opening (BBBO).[3] FUS-mediated BBBO facilitates efficient delivery of diverse therapeutics to the brain, including antibodies,[4] AAVs,[5,6] LNPs,[7,8] and polymers.[9] Its safety and efficacy have been demonstrated in both preclinical and clinical studies.[3]

Apolipoprotein E4 (*APOE4*) is the strongest genetic risk factor for Alzheimer's disease (AD), implicated in 55%–75% of AD cases.[10] *APOE4* homozygotes have a 12-fold higher risk of developing AD compared with *APOE3* carriers.[11] ApoE4 is produced predominantly by astrocytes, promoting amyloid-beta (Aβ) plaque accumulation, phosphorylated tau (pTau) pathology, neuroinflammation, and neurodegeneration.[12] Reducing apoE4 expression, therefore, represents a promising therapeutic approach for *APOE4* carriers.[5,13] Prior studies have shown that astrocyte-specific APOE4 reduction attenuates Aβ accumulation and pTau pathology, protecting against plaque-associated pathology and tau-mediated neurodegeneration.[14,15] Therefore, selective *APOE4* reduction in astrocytes provides a rational strategy for reducing apoE4 production while minimizing effects on other cell types. Current strategies to reduce brain apoE4 levels include antisense oligonucleotides (ASOs), small interfering RNA (siRNA), and monoclonal antibodies (mAbs), but these approaches typically require repeated administration.[16] In contrast, clustered regularly interspaced short palindromic repeats (CRISPR)-based genome editing offers precise, potentially one-time, and permanent therapeutic effects.[17]

We previously reported humanized *APOE4* knockdown in the mouse brain using FUS-mediated systemic delivery of CRISPR-based AAVs.[5] Here, we extend this strategy by replacing AAVs with non-viral pDNA-LNPs to establish a distinct engineering framework that accommodates larger genetic payloads and allows repeat dosing. Leveraging the larger cargo capacity of LNPs, we incorporated astrocyte-specific GfaABC1D promoter and dual guide RNAs (gRNAs) into one CRISPR plasmid, enabling efficient *APOE4* knockdown, reduction of *APOE4* mRNA and apoE4 protein expression, and decreased astrocytes and microglia activation. We selected pDNA because it enables incorporation of a cell-type-specific promoter, SaCas9, and dual gRNAs into a single programmable construct, allowing promoter-driven expression in astrocytes while maintaining compatibility with repeatable LNP administration. Although CRISPR mRNA and ribonucleoprotein (RNP) formats offer more transient expression and may reduce prolonged nuclease exposure, these formats do not provide promoter-driven cell-type-restricted expression. FUS has been used for brain delivery of ASO-LNPs for

Received 20 April 2026; accepted 13 July 2026;
https://doi.org/10.1016/j.omtn.2026.103012.

Correspondence: Kam W. Leong, Department of Biomedical Engineering, Columbia University, 3960 Broadway, New York, NY 10032, USA.
E-mail: kam.leong@columbia.edu

© 2026 The Authors.
Published by Elsevier Inc. on behalf of The American Society of Gene and Cell Therapy.
This is an open access article under the CC BY license (http://creativecommons.org/licenses/by/4.0/).

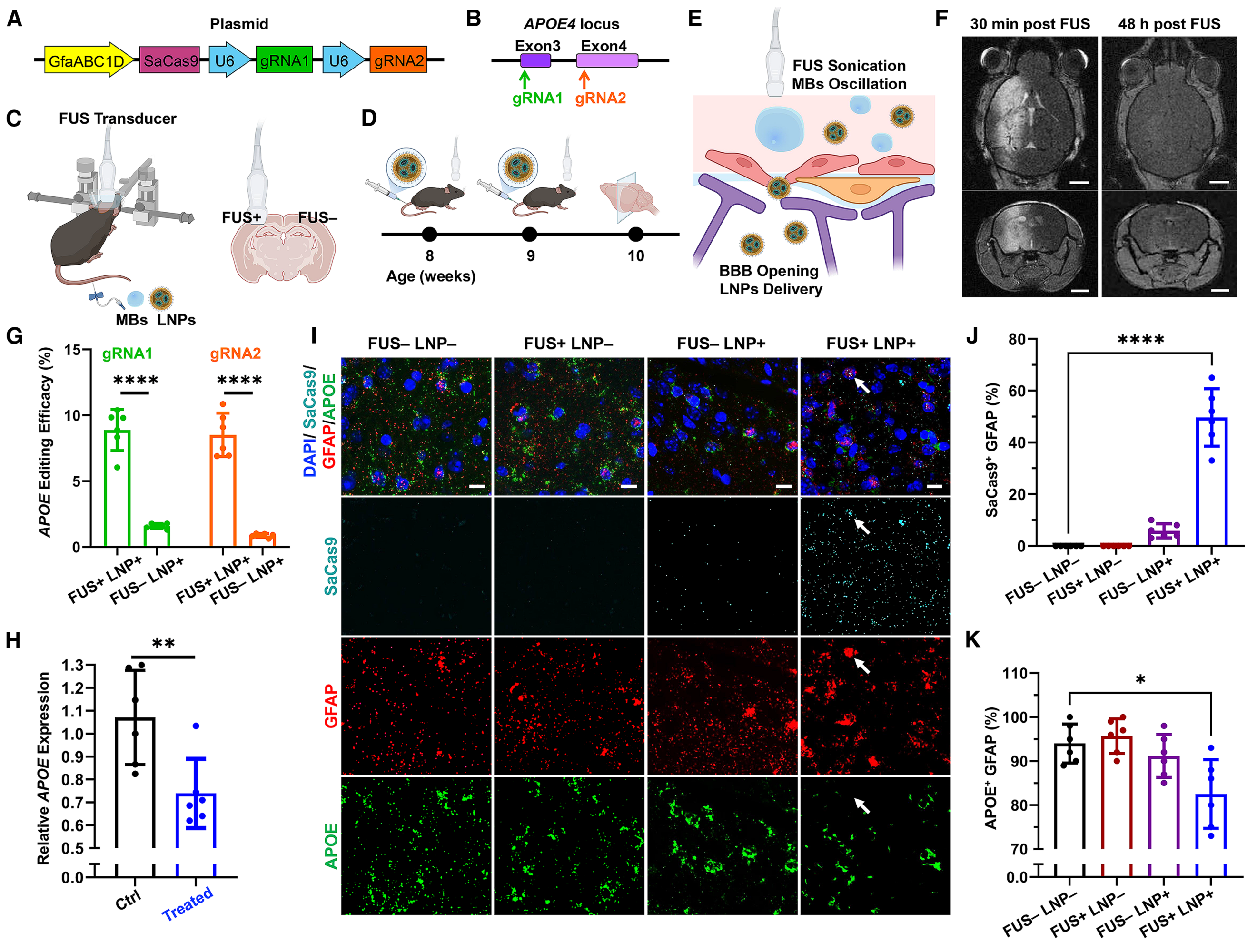

Figure 1. FUS-mediated systemic delivery of pDNA-LNPs enhanced gene editing in humanized APOE4KI mouse brains

Schematic illustrations of (A) astrocyte-targeting CRISPR plasmid containing GfaABC1D promoter, SaCas9, and dual gRNAs, (B) dual gRNAs targeting different exons of *APOE4*, (C) FUS setup, in which the left hemisphere received FUS treatment while the right hemisphere did not, (D) experimental timeline, and (E) FUS-mediated BBBO to enhance LNPs delivery. Schematics were created with BioRender. (F) Contrast-enhanced T_1-weighted MRI acquired 30 min (left) and 48 h (right) after FUS treatment in axial (top) and coronal (bottom) views. Scale bars, 2 mm. (G) Deep sequencing analysis of *APOE4* gene editing efficacy. (H) Quantification of relative *APOE4* mRNA expression in the hippocampus measured by RT-qPCR. (I) Representative confocal images of *SaCas9* (cyan), *GFAP* (red), and *APOE* (green) mRNA in the hippocampus detected by RNA ISH. Scale bars, 20 μm. Arrow indicates a $SaCas9^+APOE^-GFAP^+$ astrocyte. (J and K) Quantification of $SaCas9^+GFAP^+$ astrocytes (J) and $APOE^+GFAP^+$ astrocytes (K) relative to total $GFAP^+$ astrocytes by RNA ISH. For (G), (I), (J), and (K), "FUS-LNP-" denotes the untreated right hippocampus, "FUS+LNP-" the left hippocampus treated with FUS alone, "FUS-LNP+" the right hippocampus treated with i.v. LNPs alone, and "FUS+LNP+" the left hippocampus treated with both FUS and i.v. LNPs administration. For (H), comparisons were made between the left and right hippocampi within the same mouse, with "Ctrl" representing the ratio of left hippocampus (FUS+LNP-) to right hippocampus (FUS-LNP-), and "Treated" representing the ratio of left hippocampus (FUS+LNP+) to right hippocampus (FUS-LNP+). Data are presented as mean ± SEM (n = 6 mice per group). *$p < 0.05$; **$p < 0.01$; ****$p < 0.0001$; For (G) and (H), unpaired two-tailed Student's t test. For (J) and (K), two-way ANOVA followed by Dunnett's multiple comparisons test comparing each group with the FUS-LNP- control group.

gene regulation[7] and RNA-LNPs for protein expression;[8] however, FUS-mediated delivery of LNPs for gene editing has not been reported. This study is the first report of FUS-mediated systemic delivery of pDNA-LNPs for non-invasive, non-viral brain gene editing.

RESULTS

Since apoE protein is predominantly expressed in astrocytes, we engineered the plasmid with astrocyte-targeting promoter GfaABC1D for improved cell-type-specific expression (Figure 1A).[12,18] To enhance gene knockdown efficiency, we incorporated dual gRNAs targeting two different exons of *APOE4* in the all-in-one CRISPR pDNA for multiplexed editing (Figure 1B).[5] pDNA-LNPs were formulated at a molar ratio of D-Lin-MC3-DMA:cholesterol:DOPE:DMG-PEG2000 = 50:38.5:10:1.5 and a lipid-to-nucleic acid ratio (N:P) of 10. The synthesized pDNA-LNPs had size of 120.5 ± 4.8 nm, zeta potential of −4.1 ± 3 mV, and encapsulation efficacy >85%.

FUS was applied to the left hemisphere, with the contralateral hemisphere serving as an internal untreated control (Figure 1C). We have optimized FUS parameters and used a pulse length of 10 cycles for safe and effective BBBO.[5,19] Given the transient nature of BBBO and the repeat dosing potential of LNPs, we performed repeated treatments to improve gene editing outcomes (Figure 1D).[1,3] In the presence of intravenously administered microbubbles (MBs), FUS induces acoustic cavitation that transiently disrupts endothelial tight junctions to achieve BBBO, thereby facilitating enhanced brain delivery of LNPs (Figure 1E).[3] Contrast-enhanced T_1-weighted magnetic resonance imaging (MRI) confirmed successful BBBO in the treated left hemisphere, evidenced by enhanced gadolinium (Gd) signal (Figure 1F, left). To assess the reversibility of BBBO, Gd-based contrast agent was re-administered, followed by T_1-weighted MRI at 48 h post-treatment, which showed no signal enhancement in the previously sonicated hemisphere, thereby confirming complete restoration of BBB integrity (Figure 1F, right).[5]

Repeated FUS-mediated systemic administration of pDNA-LNPs at 0.3 mg kg^{-1} showed significant *APOE4* editing in the FUS+LNP+ hippocampus, exceeding that in the contralateral FUS-LNP+ hippocampus by more than 5-fold (Figure 1G). FUS increased gene-editing efficiencies to 8.9% and 8.5% for gRNA1 and gRNA2, respectively, compared to 1.5% and 1% in the contralateral hippocampus without FUS treatment. To further characterize the editing profiles, we quantified local insertions, deletions, and substitutions at both gRNA loci (Figure S1). These results indicate that FUS-mediated BBBO significantly enhanced the brain delivery and gene-editing efficacy of CRISPR-encoding pDNA-LNPs. This level of gene editing is comparable to that achieved by our previously reported FUS-AAV system,[5] supporting the feasibility of non-viral LNP platforms as efficient gene editing tools in the brain.

To assess the functional impact of gene editing, we analyzed *APOE4* mRNA expression. Reverse transcription-quantitative polymerase chain reaction (RT-qPCR) showed that overall *APOE4* mRNA expression in the FUS+LNP+ hippocampus was significantly reduced to 0.73 (Figures 1H and S2). Moreover, RNA *in situ* hybridization (ISH) showed that *SaCas9* mRNA was expressed in 49.6% of $GFAP^+$ astrocytes in the FUS+LNP+ hippocampus, representing the percentage of $SaCas9^+GFAP^+$ cells among total $GFAP^+$ astrocytes, compared with 4.8% in the FUS-LNP+ hippocampus (Figures 1I, 1J, and S3). Importantly, this represents a substantial improvement over our previously reported 12.9% of $SaCas9^+GFAP^+$ cells achieved using FUS-mediated systemic delivery of CRISPR-based AAVs with the hSyn promoter,[5] indicating enhanced astrocyte-targeting expression using GfaABC1D promoter. In addition, *APOE4* mRNA expression in $GFAP^+$ astrocytes was reduced to 81.5% (Figures 1I, 1K, and S3). The discrepancy between *SaCas9*-expressing astrocytes and *APOE4* knockdown may reflect that detectable *SaCas9* mRNA expression is necessary but not sufficient for productive genome editing. Final editing efficacy can be influenced by gRNA expression, Cas9 protein translation, formation of active RNP, and nuclear accumulation concentration. Unlike our previously reported AAV system, which achieved higher editing even with lower apparent *SaCas9* mRNA expression, pDNA-LNP system may produce a more transient burst of expression, which could limit cumulative editing and requires further mechanistic investigation. Notably, 61.8% of $SaCas9^+$ cells were $GFAP^+$ astrocytes (Figures S4A and S4F), further supporting astrocyte-enriched expression driven by the GfaABC1D promoter. To further define the cellular distribution of SaCas9, we also quantified $SaCas9^+$ cells among microglia, neurons, oligodendrocytes, and endothelial cells, which accounted for 19.5%, 11.5%, 8.7%, and 7.5%, respectively (Figures S4B–S4F). These results indicate that SaCas9 expression was enriched in astrocytes but not completely restricted to this population, possibly due to promoter leakiness as reported.[20] Collectively, FUS enhanced delivery of astrocyte-targeting dual gRNAs CRISPR pDNA-LNPs, leading to efficient astrocyte-enriched expression and significant *APOE4* suppression at the transcript level.

We then evaluated apoE4 protein expression following *APOE4* knockdown. Enzyme-linked immunosorbent assay (ELISA) showed that total apoE4 expression in the FUS+LNP+ hippocampus was significantly reduced to 0.71 (Figures 2A and S5). Consistently, immunofluorescence (IF) staining revealed a marked reduction of $apoE4^+$ astrocytes in FUS+LNP+ group, whereas FUS+LNP- and FUS-LNP+ groups showed little apparent change compared to the untreated FUS-LNP- group (Figure 2B). These findings indicate that repeated FUS treatment alone does not affect apoE4 expression, and that repeated LNP administration without FUS results in limited gene editing, therefore showing limited effects on apoE4 expression. Region-specific analysis demonstrated that the reduction in apoE4 expression was achieved in both the cornu ammonis (CA) and dentate gyrus (DG) regions (Figures 2C and 2D). Quantification of $apoE^+GFAP^+$ astrocytes showed reduced expression to 0.77 in CA and 0.82 in DG (Figures 2E, 2F, and S6), respectively. Together, these results demonstrate that FUS-enhanced delivery of pDNA-LNPs enabled effective suppression of apoE4 protein expression after *APOE4* knockdown in the brain.

Given that inflammatory responses remain a major concern for pDNA-LNPs, we next investigated systemic inflammation after treatment. Multiple proinflammatory cytokines increased by approximately 10-fold one day after the first and second administrations (day 1 and day 8), but returned to baseline by the following day (day 2 and day 9), and remained low at day 14, which were comparable to those in untreated APOE4KI mice and wild-type mice (Figure S7). These data indicate an acute but transient systemic immune response. Notably, FUS alone did not increase cytokine levels, whereas pDNA-LNPs alone induced a similar elevation, indicating that the inflammatory response was primarily driven by pDNA-LNPs. This transient inflammatory response is consistent with prior reports of innate immune activation after pDNA-LNP delivery, involving cGAS-STING pathway activation.[2,21] Notably, such response was observed across pDNA-LNPs containing different ionizable lipids and plasmid constructs, supporting cGAS-STING

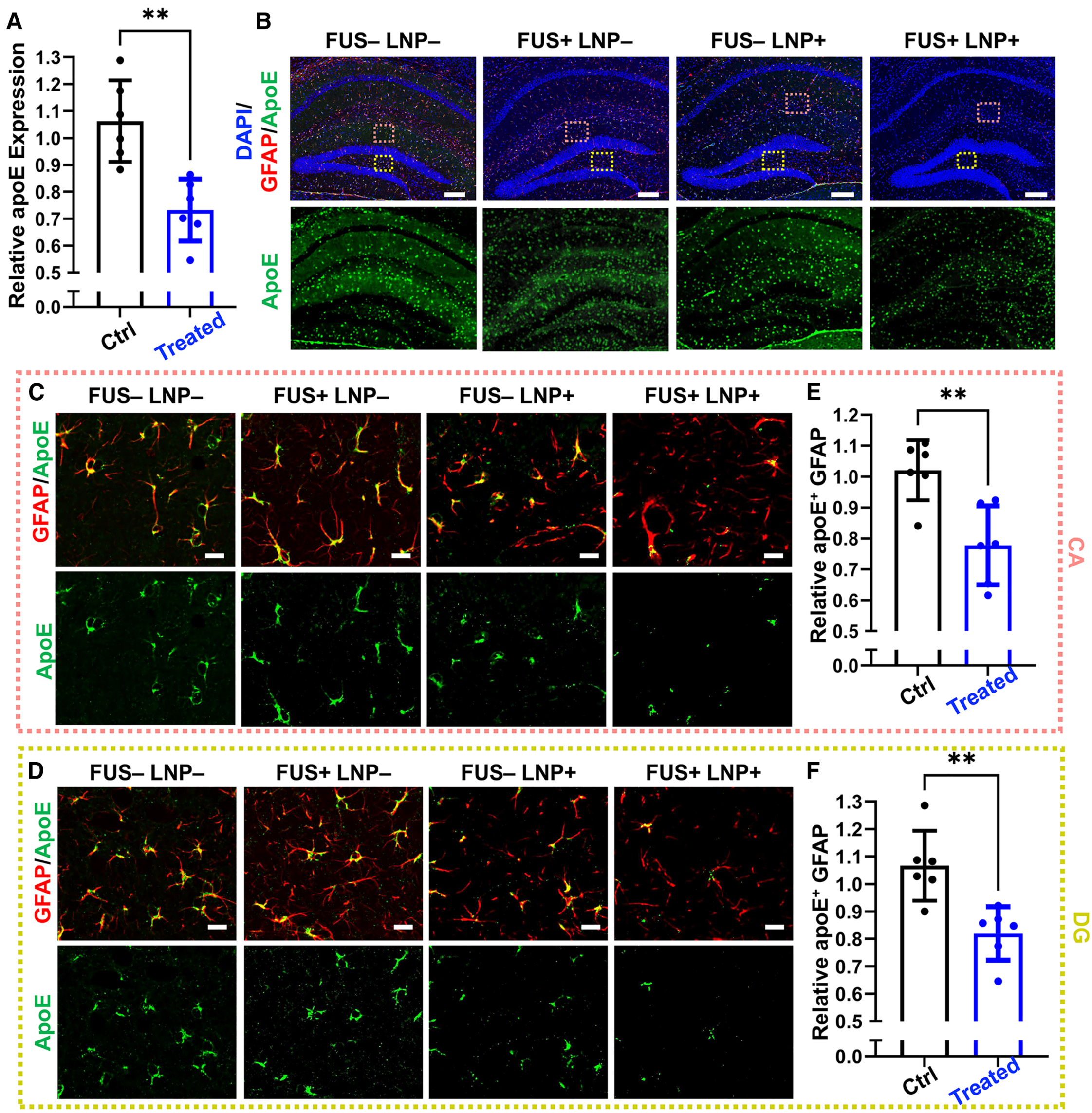

Figure 2. Decreased apoE4 protein expression following *APOE4* knockdown

(A) Quantification of relative apoE4 protein expression in the hippocampus measured by ELISA. (B) Representative confocal images of IF staining for ApoE (green), GFAP (red), and DAPI (blue) in the hippocampus. Scale bars, 200 μm. Pink and yellow boxes indicate regions of interest (ROIs) in CA (C) and DG (D) regions, respectively. Scale bars, 20 μm. (E and F) Quantification of the relative number of apoE4^{+}GFAP^{+} astrocytes in CA (E) and DG (F) regions. For (B), (C), and (D), "FUS-LNP-" denotes the untreated right hippocampus, "FUS+LNP-" the left hippocampus treated with FUS alone, "FUS-LNP+" the right hippocampus treated with i.v. LNPs alone, and "FUS+LNP+" the left hippocampus treated with both FUS and i.v. LNPs administration. For (A), (E), and (F), comparisons were made between the left and right hippocampi within the same mouse, with "Ctrl" representing the ratio of left hippocampus (FUS+LNP-) to right hippocampus (FUS-LNP-), and "treated" representing the ratio of left hippocampus (FUS+LNP+) to right hippocampus (FUS-LNP+). Data are presented as mean ± SEM (n = 6 mice per group). **$p < 0.01$; unpaired two-tailed Student's t test.

activation as a broadly shared rather than formulation-specific mechanism.[2]

Because *APOE4* is associated with astrocytes and microglia activation,[10] we also analyzed glial responses following *APOE4* knockdown. Astrocytes were evaluated by GFAP and S100β staining (Figures 3A and S8)[22] and microglia by Iba1 and CD68 staining (Figures 3F and S9).[23] For astrocytes, the number of GFAP^{+} cells remained unchanged in both CA and DG regions, but the GFAP^{+} area reduced to 0.78 in CA and 0.75 in DG (Figures 3B–3E and S10). Similarly, the S100β^{+} area reduced to 0.8 in CA and 0.83 in DG (Figures 3B–3E and S10). For microglia, both the number and area of Iba1^{+} cells reduced to 0.83 and 0.75 in CA and 0.82 and 0.74 in DG, respectively (Figures 3G–3J and S11). Consistently, the

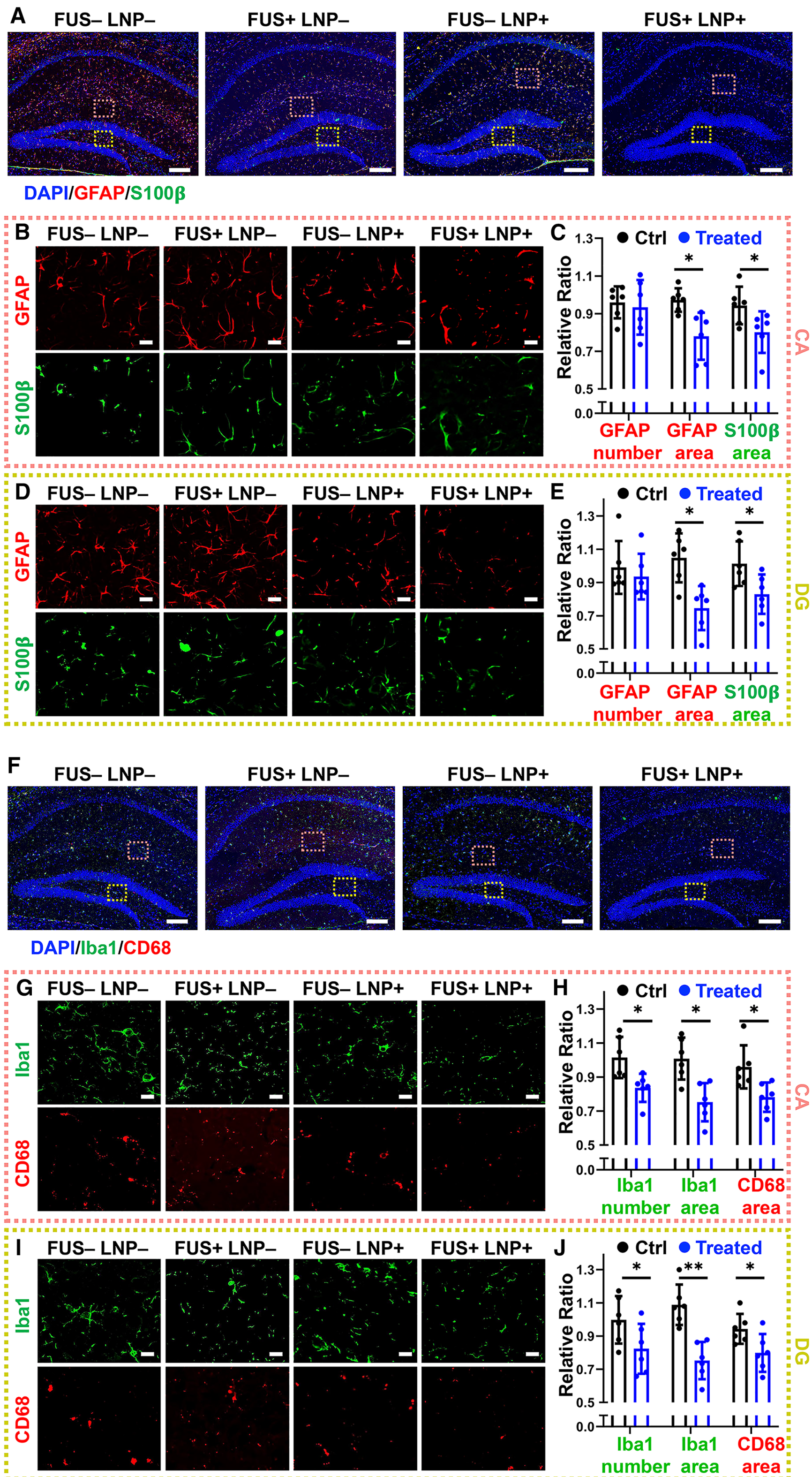

Figure 3. Decreased astrocyte and microglia activation following *APOE4* knockdown

(A) Representative confocal images of IF staining for GFAP (red), S100β (green), and DAPI (blue) in the hippocampus. Pink and yellow boxes indicate ROIs in CA (B) and DG (D) regions, respectively. (C and E) Quantification of the relative number and coverage area of GFAP, and coverage area of S100β, in CA (C) and DG (E) regions. (F) Representative confocal images of IF staining for Iba1 (green), CD68 (red), and DAPI (blue) in the hippocampus. Pink and yellow boxes indicate ROIs in CA (G) and DG (I) regions, respectively. (H and J) Quantification of the relative number and coverage area of Iba1, and coverage area of CD68, in CA (H) and DG (J) regions. Scale bars, 200 μm in (A) and (F) and 20 μm in (B), (D), (G), and (I). For (A), (B), (D), (F), (G), and (I), "FUS-LNP-" denotes the untreated right hippocampus, "FUS+LNP-" the left hippocampus treated with FUS alone, "FUS-LNP+" the right hippocampus treated with i.v. LNPs alone, and "FUS+LNP+" the left hippocampus treated with both FUS and i.v. LNPs administration. For (C), (E), (H), and (J), comparisons were made between the left and right hippocampi within the same mouse, with "Ctrl" representing the ratio of left hippocampus (FUS+LNP-) to right hippocampus (FUS-LNP-), and "Treated" representing the ratio of left hippocampus (FUS+LNP+) to right hippocampus (FUS-LNP+). Data are presented as mean ± SEM (n = 6 mice per group). $^{*}p < 0.05$; $^{**}p < 0.01$; unpaired two-tailed Student's t test.

CD68$^+$ area reduced to 0.78 in CA and 0.8 in DG (Figures 3G–3J and S11). Notably, repeated FUS-only treatment or LNPs-only treatment did not produce observable changes in astrocyte or microglia markers, which remained comparable to those observed in untreated APOE4KI and wild-type mice (Figures S12 and S13). Together, these results support that FUS-mediated pDNA-LNPs delivery significantly improved *APOE4* knockdown, leading to decreased astrocyte and microglia activation in the brain.[5,10]

DISCUSSION

In summary, we demonstrated that FUS-mediated systemic delivery of pDNA-LNPs provides a non-invasive, non-viral strategy for brain gene editing. The platform integrates three complementary components that expand the engineering design space: (1) transient and spatially controlled BBBO by FUS for non-invasive systemic delivery of therapeutics in the brain without additional carrier modification, (2) large payload capacity and repeat-dosing capability of LNPs, and (3) programmable pDNA design for incorporation of a cell-type-specific promoter and dual gRNAs CRISPR cassette to enable cell-type-specific expression and multiplex editing within a single construct. Using this approach, we achieved efficient *APOE4* disruption, accompanied by reductions in transcript, protein, and glial activation. However, the threshold of *APOE4* reduction or glia modulation required for therapeutic benefit remains uncertain and may depend on disease stage and intervention timing. Prior studies suggest that 50% reduction of apoE4 is well tolerated in mice, and ASO-mediated reduction of apoE4 by approximately 40%–50% reduced Aβ deposition, Aβ-associated neuritic dystrophy, tau pathology, neurodegeneration, and neuroinflammation.[13,24,25] Future investigations using more advanced transgenic AD-relevant mouse models, such as amyloid- and tau-bearing *APOE4* mice, together with longer treatment windows and multiple dosing regimens, will be important to determine the editing and glial modulation thresholds required for sustained therapeutic benefit. An additional translational consideration is that non-allele-selective *APOE* disruption may reduce not only apoE4 but also protective or neutral apoE isoforms in *APOE3*/*APOE4* and *APOE2*/*APOE4* heterozygotes. Because apoE contributes to lipid transport, synaptic maintenance, neuronal homeostasis, and immune regulation, excessive or broad *APOE* depletion may have adverse effects.[13] Future therapeutic development may therefore require *APOE4*-to-*APOE3* or *APOE4*-to-*APOE2* conversion through base editing, or dosing strategies that achieve partial *APOE* reduction while preserving essential apoE function.

The therapeutic promise of pDNA-LNPs is substantially enhanced by FUS-mediated targeted, transient, and non-invasive BBBO. Such spatial control is particularly crucial for neurological disease, where pathology is often regionally heterogeneous and therapeutic benefit may depend on restricting activity to affected regions while minimizing exposure in unaffected regions. Moreover, FUS overcomes key limitations of direct parenchymal injection, which is invasive and spatially restricted, therefore supporting treatment of diffuse neurodegenerative diseases such as AD. By enhancing targeted delivery efficiency, FUS may reduce the amount of therapeutics required, potentially improving tolerability and minimizing side effects. Supported by growing clinical experience with FUS-mediated BBBO,[4,26] the synergy of FUS technology with LNP-based gene therapy provides a practical path toward non-viral, non-invasive treatment of brain diseases.

Compared with viral vectors, LNPs offer several advantages. Their larger payload capability can accommodate more complex gene-editing tools, thereby avoiding split vector approaches and the associated increases in manufacturing complexity and reductions in co-delivery efficiency. Their low immunogenicity supports repeat administration, which is valuable for personalized treatment and diseases requiring iterative intervention. Moreover, LNPs offer a modular formulation framework in which payloads can be readily exchanged, facilitating rapid product development as demonstrated by the mRNA vaccines against SARS-CoV-2, while ongoing advances in formulation and functionalization are expected to improve tissue tropism and cellular uptake.[1] Although FUS-mediated brain delivery of CRISPR pDNA-LNPs has not been reported, a previous report used FUS to enhance brain delivery of polymeric NPs carrying Cas9 mRNA and gRNA for gene editing in Ai9 mice.[9] Compared with long-circulating polymeric NPs, LNPs have different circulation stability, serum interactions, and cell-tropism constraints.[1] Systemic comparison among AAVs, LNPs, and polymeric NPs for gene editing would be valuable for defining the relative advantages, limitations, and optimal application scenarios of each delivery platform.

Payload format is also an important consideration for non-viral gene editing. pDNA enables promoter-driven cell-type-specific expression but holds innate immune activation and may also cause prolonged expression, thereby increasing the potential for off-target editing and genotoxicity concerns. pDNA requires transcription and translation into mRNA and gRNA to form the active RNP, whereas mRNA bypasses transcription, and RNP bypasses both steps. CRISPR mRNA/gRNA provides more transient nuclease expression to reduce risks associated with prolonged Cas9 activity, while RNP offers the shortest editing window to reduce off-target activity. However, neither format supports promoter-driven cell-type-specific expression. Moreover, productive editing with mRNA-based systems requires efficient and coordinated intracellular delivery of both Cas9 mRNA and gRNA. RNP delivery poses additional formulation challenges because of the large molecular size and complex electrostatic properties. Future studies should define the duration of expression after pDNA-LNPs delivery and compare with mRNA/gRNA and RNP payloads to optimize the balance among editing efficiency, cell-type selectivity, expression duration, and safety.

Regarding the inflammation response after FUS-mediated pDNA-LNP delivery, plasma cytokine measurements provide useful information about systemic immune activation but do not reflect local inflammatory responses after FUS-mediated BBBO. Prior studies have reported transient local neuroinflammatory responses after FUS-mediated BBBO, including astrogliosis, microgliosis, cytokine induction, and immune-cell infiltration, with the magnitude and duration

depending on acoustic pressure, pulse sequence, microbubble dose, and so on.[3] Our previous studies using optimized FUS parameters showed that local inflammatory responses after BBBO were transient and typically resolved within days.[27,28] Prior work in rat models using MRI-guided FUS with systemically administered MBs showed that most local inflammatory responses lasted 24 h, similar to observations in mouse models.[29] In contrast, a study using surgically implanted FUS transducer without MBs showed local inflammatory responses lasting up to 15 days, highlighting the substantial influence of FUS configuration and BBBO methodology.[30]

Our findings position FUS-mediated pDNA-LNP delivery as a distinct and modular platform for non-invasive, non-viral, region- and cell-type- specific brain gene editing. The design principle could be adapted to other genomic targets and complex gene-editing tools. Such modularity is particularly important for neurodegenerative disorders, where therapeutic efficacy may depend on regional and cell-type selectivity, repeated intervention, and the ability to incorporate complex therapeutic cassettes. Future advances will likely come from optimization of LNP composition to improve endosomal escape and nuclear entry, enhance tissue-specific and cell-type-specific delivery, and mitigate innate immune activation.[1,2] Additional investigation of redosing interval, editing durability, and safety will be helpful to define the optimal therapeutic window and assess the translational potential of this platform.

MATERIALS AND METHODS

pDNA design

The pX601-AAV-CMV::NLS-SaCas9-NLS-3xHA-bGHpA;U6::BsaI-sgRNA vector (Addgene plasmid #61591) was used as the backbone. Cytomegalovirus (CMV) promoter was replaced with the GfaABC1D promoter for astrocyte-enriched expression.[18] An additional gRNA scaffold was inserted into the vector to generate dual U6 promoter-driven gRNAs. Two *APOE*-targeting gRNAs were selected based on our previous report, with gRNA1 targeting exon 3 (sequence: 5′-GGCCAAGGTGGAGCAAGCGG-3′) and gRNA2 targeting exon 4 (sequence: 5′-GGCCTACAAATCGGAACTGG-3′).[5] Notably, the gRNAs are not *APOE4*-specific. Oligonucleotides were synthesized by Integrated DNA Technologies (IDT). The plasmid was transformed into New England Biolabs (NEB) 5-alpha competent *E. coli* according to the manufacturer's protocol. Endotoxin-free plasmid was purified using the NucleoBond Xtra Midi Plus EF kit (Macherey-Nagel) according to the manufacturer's instructions. The final construct was verified by whole-plasmid sequencing (Plasmidsaurus).

Synthesis of pDNA-LNPs

D-Lin-MC3-DMA and DOPE (1,2-dioleoyl-sn-glycero-3-phosphoethanolamine) were purchased from MedChemExpress, cholesterol was purchased from Sigma-Aldrich, and DMG-PEG 2000 (1,2-dimyristoyl-rac-glycero-3-methoxypolyethylene glycol-2000) was purchased from Avanti Polar Lipids. LNPs were formulated at a molar ratio of 50% D-Lin-MC3-DMA, 38.5% cholesterol, 10% DOPE, and 1.5% DMG-PEG 2000. Lipids were dissolved in ethanol and mixed with an aqueous phase containing pDNA in 50 mM citrate buffer (pH 4) at an ethanol-to-aqueous volume ratio of 1:3 (v/v) and a lipid-to-nucleic acid (N:P) ratio of 10. The pDNA-LNPs were incubated at room temperature for 15 min and then dialyzed against 1× PBS using Pur-A-Lyzer Midi Dialysis Kits (MWCO 3.5 kDa; Sigma-Aldrich).

FUS-mediated BBBO for pDNA-LNP delivery

The ultrasound system used in this study was developed as previously described.[5,6,19] FUS was delivered using a P4-1 phased-array transducer (96 elements, 2.5 MHz center frequency, 1.5–3.5 MHz—6 dB bandwidth; ATL, Philips), which was controlled by a 3D positioning system (VXM, Velmex Inc.) and operated with a research ultrasound system (Vantage 256, Verasonics Inc.). A custom MATLAB script was used to generate rapid bursts of short pulses at a transmit frequency of 1.5 MHz and a pulse length of 10 cycles. The focal depth was 35 mm from the transducer aperture. Each treatment session lasted 2 min, during which 60 bursts were delivered at a burst repetition frequency (BRF) of 0.5 Hz. Each burst consisted of 100 focused pulses transmitted at a pulse repetition frequency (PRF) of 1,000 Hz.

For FUS treatment, 8-week-old adult mice were anesthetized with 3%–4% isoflurane, and adequate anesthesia was confirmed by the absence of a toe-pinch withdrawal reflex. Each mouse was placed on a heating pad with the head secured in a stereotactic frame (David Kopf Instruments) under continuous anesthesia. After the scalp was shaved and depilated, degassed ultrasound gel was applied to the head for acoustic coupling, and a water bath filled with degassed deionized water was positioned above the head. To target the region of interest, the intersection of the lambdoid and sagittal sutures (lambda) was identified through the intact scalp, and a metal crosshair was placed on top to align the lateral and elevational axes of the transducer. The transducer was then submerged in the water bath, and B-mode imaging at 2.5 MHz was used to position the focus over the left hemisphere, 3 mm anterior and 2 mm lateral to lambda. The right hemisphere served as the untreated control. After alignment, the metal crosshair was removed.

Polydisperse MBs were synthesized in-house and activated according to previously published protocols.[5,19] Immediately before sonication, MBs were diluted in 100 μL of sterile saline to a final concentration of 8×10^8 MB/mL. The left hemisphere was first sonicated for 10 s to obtain a baseline acoustic signal. Sonication was then initiated, followed by intravenous (i.v.) administration of MBs via the lateral tail vein using a 27G winged infusion set. Subsequently, freshly prepared pDNA-LNPs were administered intravenously at a dose of 0.3 mg kg^{-1}. In the control group, mice received MBs only without pDNA-LNPs. Sonication was continued for 2 min after administration of MBs. Upon completion of the procedure, the mouse was released from the stereotactic frame and monitored until full recovery from anesthesia. Each group consisted of six mice, including three males and three females.

To confirm BBBO, 0.2 mL of the Gd-based contrast agent Omniscan (GE Healthcare) was administered intraperitoneally 30 min after the

FUS procedure. The mouse was anesthetized and positioned in a 30 mm birdcage coil for imaging using a 9.4T MRI system (Bruker Medical). Contrast-enhanced T1-weighted 2D FLASH images were acquired in the axial and coronal planes (TR/TE, 230/3.3 ms; flip angle, 70°; averages, 6; field of view, 25.6 × 25.6 mm^2; resolution, 100 × 100 × 400 μm^3). To confirm restoration of BBB integrity after BBBO, 0.2 mL of Gd-based contrast agent was re-administered 48 h after FUS treatment, followed by T1-weighted MRI under the same conditions.

The second treatment was performed one week after the initial session using the same protocol. One week after the second treatment, mice were anesthetized and euthanized by transcardial perfusion with cold 1× PBS followed by cold 4% paraformaldehyde (PFA) in PBS. All animal studies were performed in accordance with protocols AC-AABU2665, AC-AABG4559, and AC-AABX4654 approved by the Columbia University Institutional Animal Care and Use Committee (IACUC).

DATA AND CODE AVAILABILITY

The data supporting the findings of this study are available within the article and its supplemental information.

ACKNOWLEDGMENTS

This work was supported by Columbia University Alzheimer's Disease Research Center Research Education Component (K.Z., P30AG066462, PI: Small Scott, MD), and Career Development Award from the American Society of Gene & Cell Therapy in partnership with the Focused Ultrasound Foundation (K.Z., CDA25-009-FUSF). The content is solely the responsibility of the authors and does not necessarily represent the official views of the American Society of Gene & Cell Therapy or the Focused Ultrasound Foundation. This study used the Confocal and Specialized Microscopy Shared Resource of the Herbert Irving Comprehensive Cancer Center at Columbia University, funded in part through NIH/NCI Cancer Center Support Grant P30CA013696.

AUTHOR CONTRIBUTIONS

K.Z. and K.W.L. designed the research; K.Z. and F.N.T. conducted the experiments; K.Z. analyzed data; and K.Z., K.W.L., F.N.T., and E.E.K., wrote the paper.

DECLARATION OF INTERESTS

The authors declare no competing interests.

SUPPLEMENTAL INFORMATION

Supplemental information can be found online at https://doi.org/10.1016/j.omtn.2026.103012.

REFERENCES

1. Raguram, A., Banskota, S., and Liu, D.R. (2022). Therapeutic in vivo delivery of gene editing agents. Cell *185*, 2806–2827. https://doi.org/10.1016/j.cell.2022.03.045.

2. Patel, M.N., Tiwari, S., Wang, Y., O'Neill, S., Wu, J., Omo-Lamai, S., Espy, C., Chase, L.S., Majumder, A., Hoffman, E., et al. (2026). Safer non-viral DNA delivery using lipid nanoparticles loaded with endogenous anti-inflammatory lipids. Nat. Biotechnol. *44*, 79–89. https://doi.org/10.1038/s41587-025-02556-5.

3. Gorick, C.M., Breza, V.R., Nowak, K.M., Cheng, V.W.T., Fisher, D.G., Debski, A.C., Hoch, M.R., Demir, Z.E.F., Tran, N.M., Schwartz, M.R., et al. (2022). Applications of focused ultrasound-mediated blood-brain barrier opening. Adv. Drug Deliv. Rev. *191*, 114583. https://doi.org/10.1016/j.addr.2022.114583.

4. Rezai, A.R., D'Haese, P.-F., Finomore, V., Carpenter, J., Ranjan, M., Wilhelmsen, K., Mehta, R.I., Wang, P., Najib, U., Vieira Ligo Teixeira, C., et al. (2024). Ultrasound blood–brain barrier opening and aducanumab in Alzheimer's disease. N. Engl. J. Med. *390*, 55–62. https://doi.org/10.1056/NEJMoa2308719.

5. Zheng, K., Tsitsos, F.N., Batts, A.J., Ji, R., Nuriel, T., Konofagou, E.E., and Leong, K.W. (2025). Focused ultrasound-mediated APOE4 knockdown in mouse brain. Alzheimer's Dement. *21*, e70464. https://doi.org/10.1002/alz.70464.

6. Lao, Y.-H., Ji, R., Zhou, J.K., Snow, K.J., Kwon, N., Saville, E., He, S., Chauhan, S., Chi, C.-W., Datta, M.S., et al. (2023). Focused ultrasound–mediated brain genome editing. Proc. Natl. Acad. Sci. USA *120*, e2302910120. https://doi.org/10.1073/pnas.2302910120.

7. Ediriweera, G.R., Sivaram, A.J., Cowin, G., Brown, M.L., Mcalary, L., Lum, J.S., Fletcher, N.L., Robinson, L., Simpson, J.D., Chen, L., et al. (2025). Lipid nanoparticles and transcranial focused ultrasound enhance the delivery of SOD1 antisense oligonucleotides to the murine brain for ALS therapy. J. Contr. Release *378*, 221–235. https://doi.org/10.1016/j.jconrel.2024.11.074.

8. Shumer-Elbaz, M., Ad-El, N., Chulanova, Y., Brier, D., Goldsmith, M., Bismuth, M., Brosque, A., Gattegno, R., Sher, D., Gutkin, A., et al. (2025). Low-frequency ultrasound-mediated blood-brain barrier opening enables non-invasive lipid nanoparticle RNA delivery to glioblastoma. J. Contr. Release *385*, 114018. https://doi.org/10.1016/j.jconrel.2025.114018.

9. Kwak, G., Grewal, A., Slika, H., Mess, G., Li, H., Kwatra, M., Poulopoulos, A., Woodworth, G.F., Eberhart, C.G., Ko, H.S., et al. (2024). Brain nucleic acid delivery and genome editing via focused ultrasound-mediated blood-brain barrier opening and long-circulating nanoparticles. ACS Nano *18*, 24139–24153. https://doi.org/10.1021/acsnano.4c05270.

10. Blumenfeld, J., Yip, O., Kim, M.J., and Huang, Y. (2024). Cell type-specific roles of APOE4 in Alzheimer disease. Nat. Rev. Neurosci. *25*, 91–110. https://doi.org/10.1038/s41583-023-00776-9.

11. Serrano-Pozo, A., Das, S., and Hyman, B.T. (2021). APOE and Alzheimer's disease: advances in genetics, pathophysiology, and therapeutic approaches. Lancet Neurol. *20*, 68–80. https://doi.org/10.1016/S1474-4422(20)30412-9.

12. Yamazaki, Y., Zhao, N., Caulfield, T.R., Liu, C.-C., and Bu, G. (2019). Apolipoprotein E and Alzheimer disease: pathobiology and targeting strategies. Nat. Rev. Neurol. *15*, 501–518. https://doi.org/10.1038/s41582-019-0228-7.

13. Vance, J.M., Farrer, L.A., Huang, Y., Cruchaga, C., Hyman, B.T., Pericak-Vance, M.A., Goate, A.M., Greicius, M.D., Griswold, A.J., Haines, J.L., et al. (2024). Report of the APOE4 National Institute on Aging/Alzheimer disease sequencing project consortium working group: reducing APOE4 in carriers is a therapeutic goal for Alzheimer's disease. Ann. Neurol. *95*, 625–634. https://doi.org/10.1002/ana.26864.

14. Wang, C., Xiong, M., Gratuze, M., Bao, X., Shi, Y., Andhey, P.S., Manis, M., Schroeder, C., Yin, Z., Madore, C., et al. (2021). Selective removal of astrocytic APOE4 strongly protects against tau-mediated neurodegeneration and decreases synaptic phagocytosis by microglia. Neuron *109*, 1657–1674.e7. https://doi.org/10.1016/j.neuron.2021.03.024.

15. Mahan, T.E., Wang, C., Bao, X., Choudhury, A., Ulrich, J.D., and Holtzman, D.M. (2022). Selective reduction of astrocyte apoE3 and apoE4 strongly reduces Aβ accumulation and plaque-related pathology in a mouse model of amyloidosis. Mol. Neurodegener. *17*, 13. https://doi.org/10.1186/s13024-022-00516-0.

16. Jackson, R.J., Hyman, B.T., and Serrano-Pozo, A. (2024). Multifaceted roles of APOE in Alzheimer disease. Nat. Rev. Neurol. *20*, 457–474. https://doi.org/10.1038/s41582-024-00988-2.

17. Cong, L., Ran, F.A., Cox, D., Lin, S., Barretto, R., Habib, N., Hsu, P.D., Wu, X., Jiang, W., Marraffini, L.A., and Zhang, F. (2013). Multiplex genome engineering using CRISPR/Cas systems. Science *339*, 819–823. https://doi.org/10.1126/science.1231143.

18. Gleichman, A.J., Kawaguchi, R., Sofroniew, M.V., and Carmichael, S.T. (2023). A toolbox of astrocyte-specific, serotype-independent adeno-associated viral vectors using microRNA targeting sequences. Nat. Commun. *14*, 7426. https://doi.org/10.1038/s41467-023-42746-w.

19. Tsitsos, F.N., Batts, A.J., Jimenez, D.A., Macsemchuk, C.A., Li, C., Ji, R., Bae, S., Ciaccio, G.L., Theodorou, A., Ramirez, R.K., et al. (2025). Characterization of microbubble cavitation in theranostic ultrasound-mediated blood-brain barrier opening

for gene delivery. J. Contr. Release *385*, 113986. https://doi.org/10.1016/j.jconrel.2025.113986.

20. Taschenberger, G., Tereshchenko, J., and Kügler, S. (2017). A microRNA124 target sequence restores astrocyte specificity of gfaABC1D-driven transgene expression in AAV-mediated gene transfer. Mol. Ther. Nucleic Acids *8*, 13–25. https://doi.org/10.1016/j.omtn.2017.03.009.

21. Tursi, N.J., Tiwari, S., Bedanova, N., Kannan, T., Parzych, E., Okba, N., Liaw, K., Sárközy, A., Livingston, C., Trullen, M.I., et al. (2025). Modulation of lipid nanoparticle-formulated plasmid DNA drives innate immune activation promoting adaptive immunity. Cell Rep. Med. *6*, 102035. https://doi.org/10.1016/j.xcrm.2025.102035.

22. Escartin, C., Galea, E., Lakatos, A., O'Callaghan, J.P., Petzold, G.C., Serrano-Pozo, A., Steinhäuser, C., Volterra, A., Carmignoto, G., Agarwal, A., et al. (2021). Reactive astrocyte nomenclature, definitions, and future directions. Nat. Neurosci. *24*, 312–325. https://doi.org/10.1038/s41593-020-00783-4.

23. Lier, J., Streit, W.J., and Bechmann, I. (2021). Beyond activation: characterizing microglial functional phenotypes. Cells *10*, 2236. https://doi.org/10.3390/cells10092236.

24. Litvinchuk, A., Huynh, T.P.V., Shi, Y., Jackson, R.J., Finn, M.B., Manis, M., Francis, C.M., Tran, A.C., Sullivan, P.M., Ulrich, J.D., et al. (2021). Apolipoprotein E4 reduction with antisense oligonucleotides decreases neurodegeneration in a tauopathy model. Ann. Neurol. *89*, 952–966. https://doi.org/10.1002/ana.26043.

25. Huynh, T.-P.V., Liao, F., Francis, C.M., Robinson, G.O., Serrano, J.R., Jiang, H., Roh, J., Finn, M.B., Sullivan, P.M., Esparza, T.J., et al. (2017). Age-dependent effects of apoE reduction using antisense oligonucleotides in a model of β-amyloidosis. Neuron *96*, 1013–1023.e4. https://doi.org/10.1016/j.neuron.2017.11.014.

26. Bae, S., Liu, K., Pouliopoulos, A.N., Ji, R., Jiménez-Gambín, S., Yousefian, O., Kline-Schoder, A.R., Batts, A.J., Tsitsos, F.N., Kokossis, D., et al. (2024). Transcranial blood-brain barrier opening in Alzheimer's disease patients using a portable focused ultrasound system with real-time 2-D cavitation mapping. Theranostics *14*, 4519–4535. https://doi.org/10.7150/thno.94206.

27. Kline-Schoder, A.R., Chintamen, S., Willner, M.J., DiBenedetto, M.R., Noel, R.L., Batts, A.J., Kwon, N., Zacharoulis, S., Wu, C.-C., Menon, V., et al. (2024). Characterization of the responses of brain macrophages to focused ultrasound-mediated blood–brain barrier opening. Nat. Biomed. Eng. *8*, 650–663. https://doi.org/10.1038/s41551-023-01107-0.

28. Ji, R., Karakatsani, M.E., Burgess, M., Smith, M., Murillo, M.F., and Konofagou, E.E. (2021). Cavitation-modulated inflammatory response following focused ultrasound blood-brain barrier opening. J. Contr. Release *337*, 458–471. https://doi.org/10.1016/j.jconrel.2021.07.042.

29. Kovacs, Z.I., Kim, S., Jikaria, N., Qureshi, F., Milo, B., Lewis, B.K., Bresler, M., Burks, S.R., and Frank, J.A. (2017). Disrupting the blood–brain barrier by focused ultrasound induces sterile inflammation. Proc. Natl. Acad. Sci. USA *114*, E75–E84. https://doi.org/10.1073/pnas.1614777114.

30. Mascotte-Cruz, J.U., Vera, A., Leija, L., Lopez-Salas, F.E., Gradzielski, M., Koetz, J., Gatica-García, B., Rodríguez-Oviedo, C.P., Valenzuela-Arzeta, I.E., Escobedo, L., et al. (2024). Focused ultrasound on the substantia nigra enables safe neurotensin-polyplex nanoparticle-mediated gene delivery to dopaminergic neurons intranasally and by blood circulation. Discov. Nano *19*, 60. https://doi.org/10.1186/s11671-024-04005-9.

OMTN, Volume *37*

Supplemental information

Focused ultrasound-mediated lipid nanoparticle delivery for brain gene editing

Kaiyuan Zheng, Fotios N. Tsitsos, Elisa E. Konofagou, and Kam W. Leong

SUPPLEMENTAL FIGURES

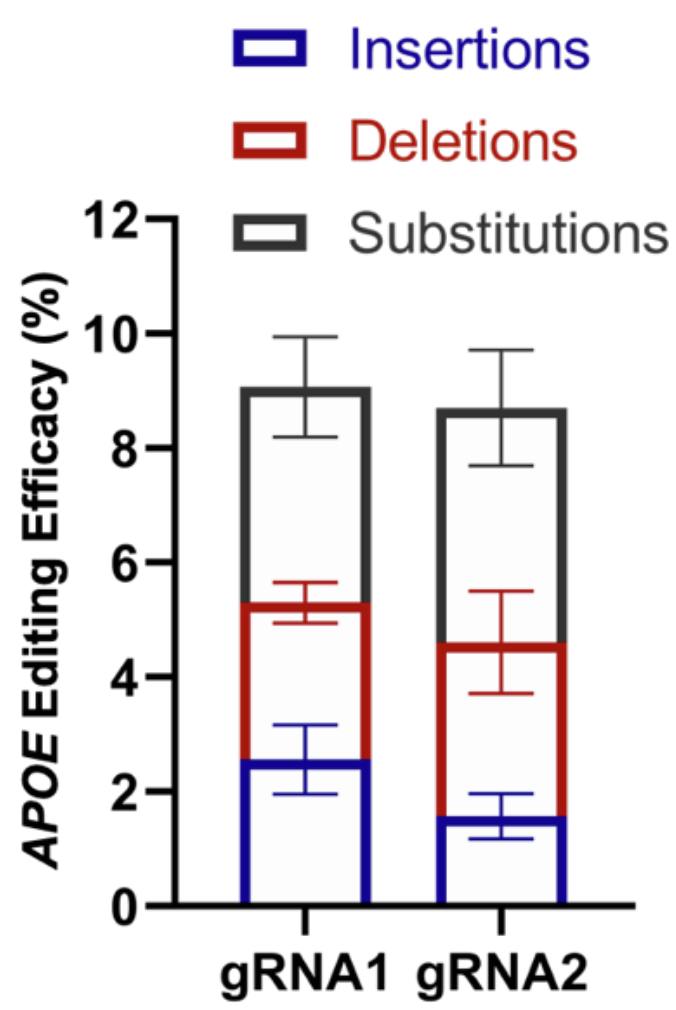

Figure S1. *APOE4* editing profiles at two gRNA target sites. Stacked bars show the percentage of insertions, deletions, and substitutions contributing to total editing efficacy at each target site.

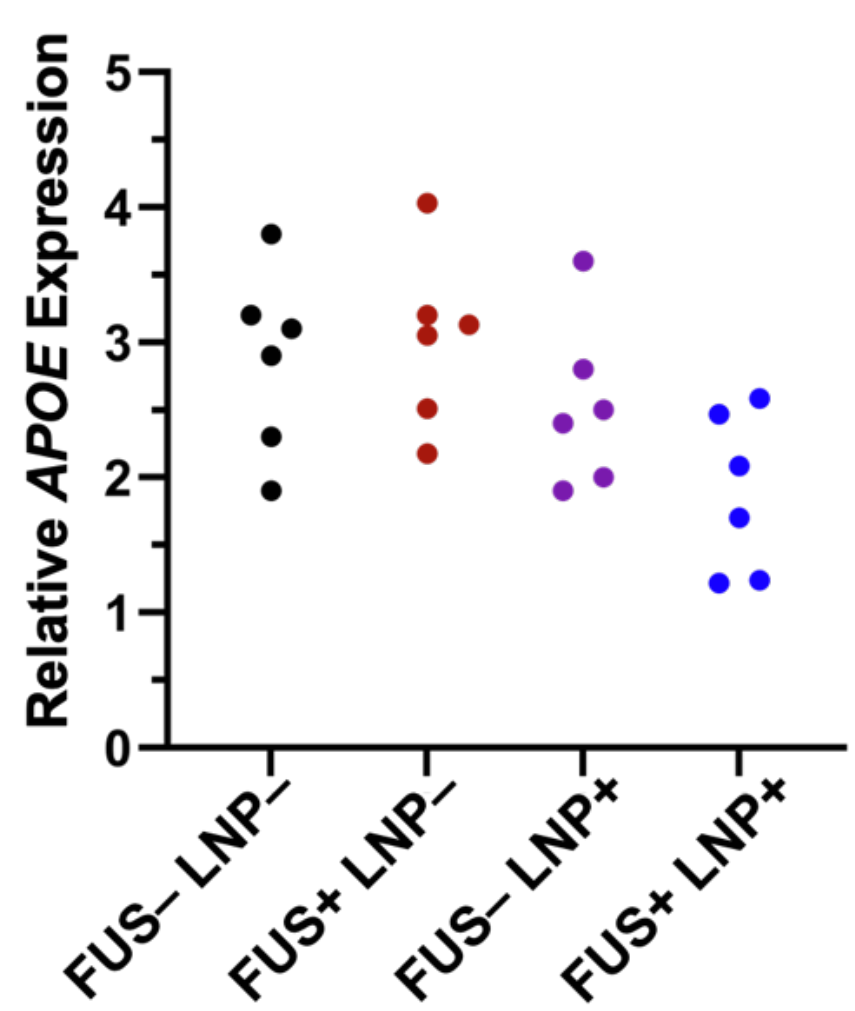

Figure S2. Raw values used to quantify relative *APOE* mRNA expression levels in the hippocampus by RT-qPCR.

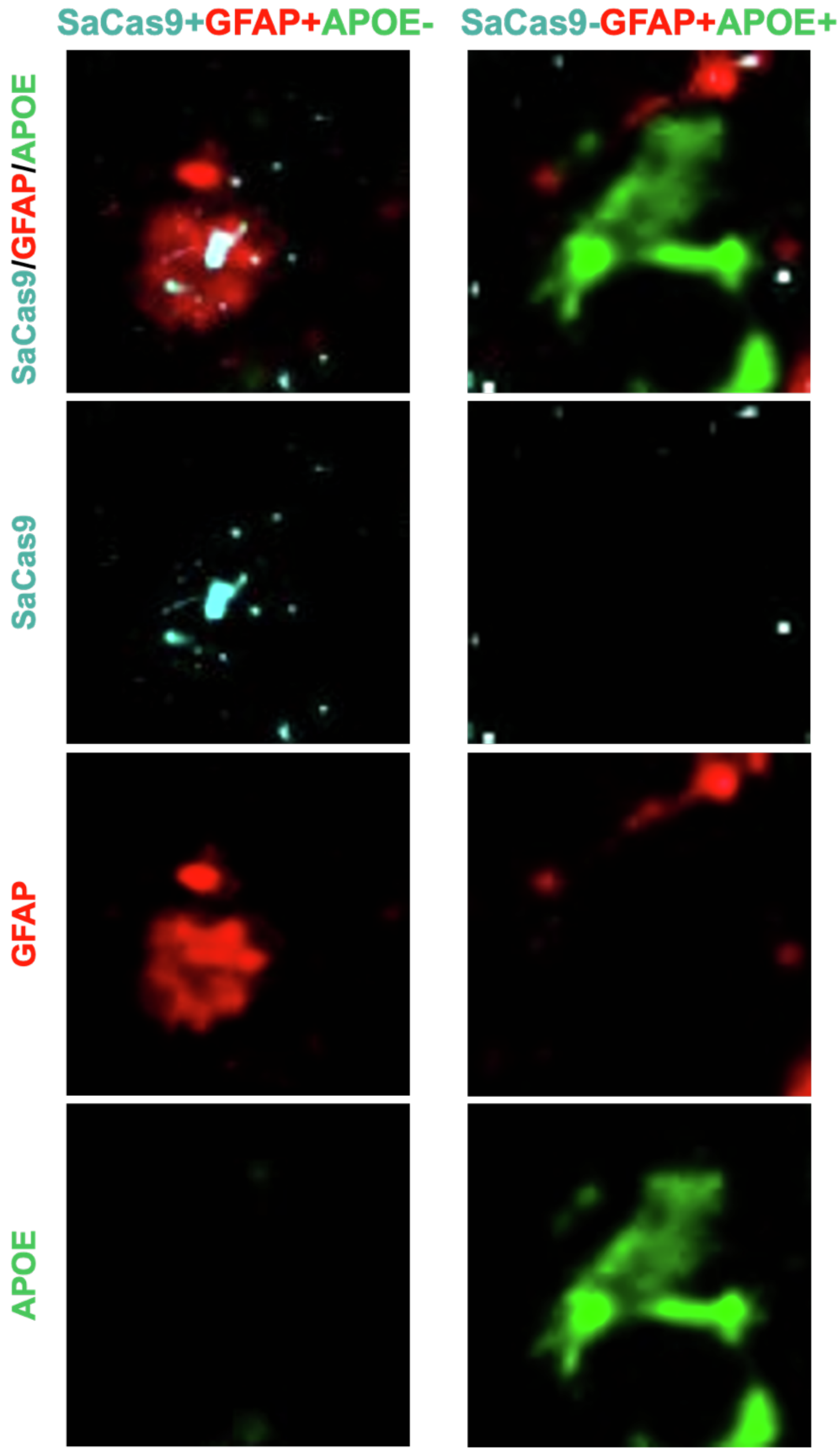

Figure S3. Enlarged representative confocal images showing *SaCas9*$^{+}$*GFAP*$^{+}$*APOE*$^{-}$ cell and *SaCas9*$^{-}$*GFAP*$^{+}$*APOE*$^{+}$ cell detected by RNA ISH.

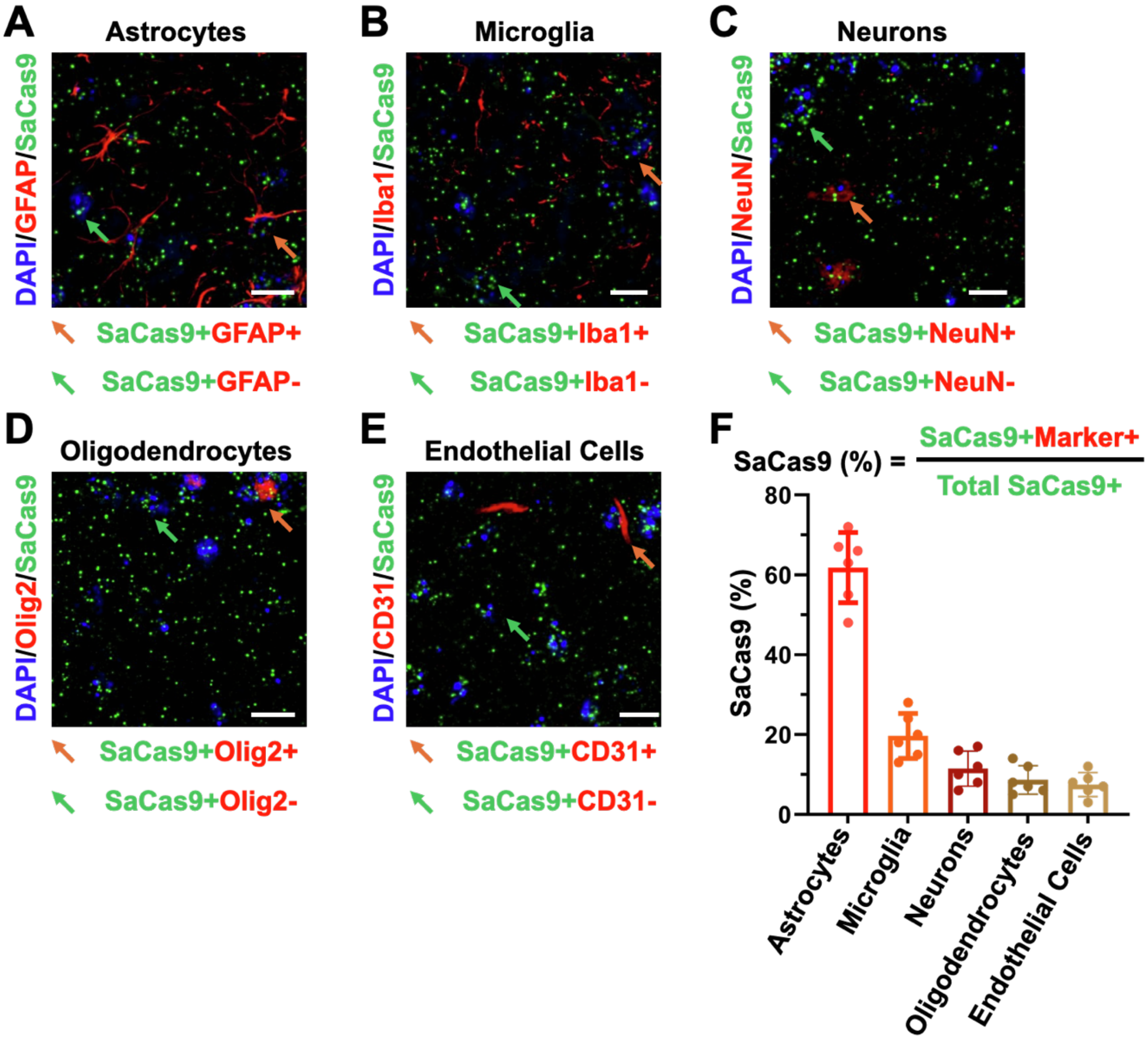

Figure S4. Representative confocal images showing *SaCas9* mRNA (green) distribution in hippocampal astrocytes stained for GFAP (A, red), microglia stained for Iba1 (B, red), neurons stained for NeuN (C, red), oligodendrocytes stained for Olig2 (D, red), and endothelial cells stained for CD31 (E, red). Scale bars, 20 μm. (F) Quantification of *SaCas9* mRNA distribution across different cell types.

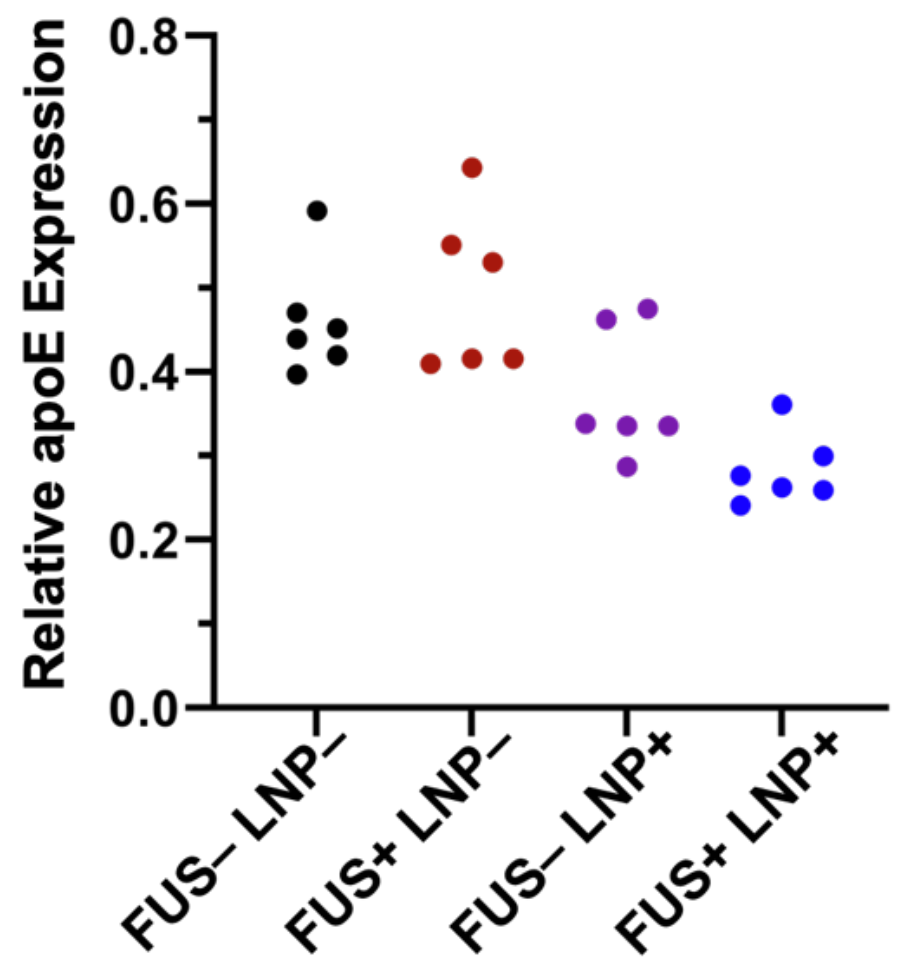

Figure S5. Raw values used to quantify relative apoE4 protein expression levels in the hippocampus by ELISA.

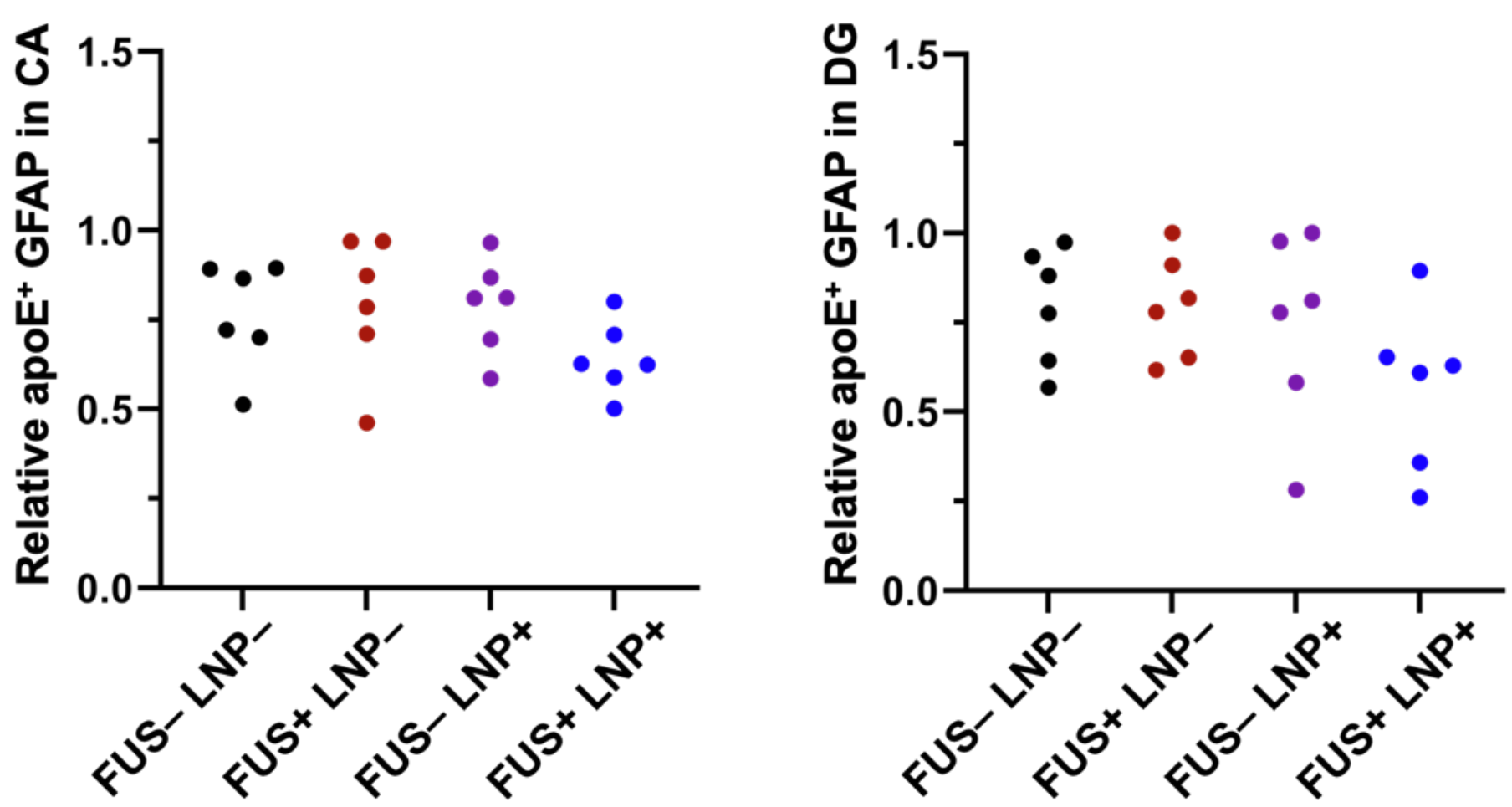

Figure S6. Raw values used to quantify apoE4-positive GFAP-staining astrocytes in the CA and DG regions.

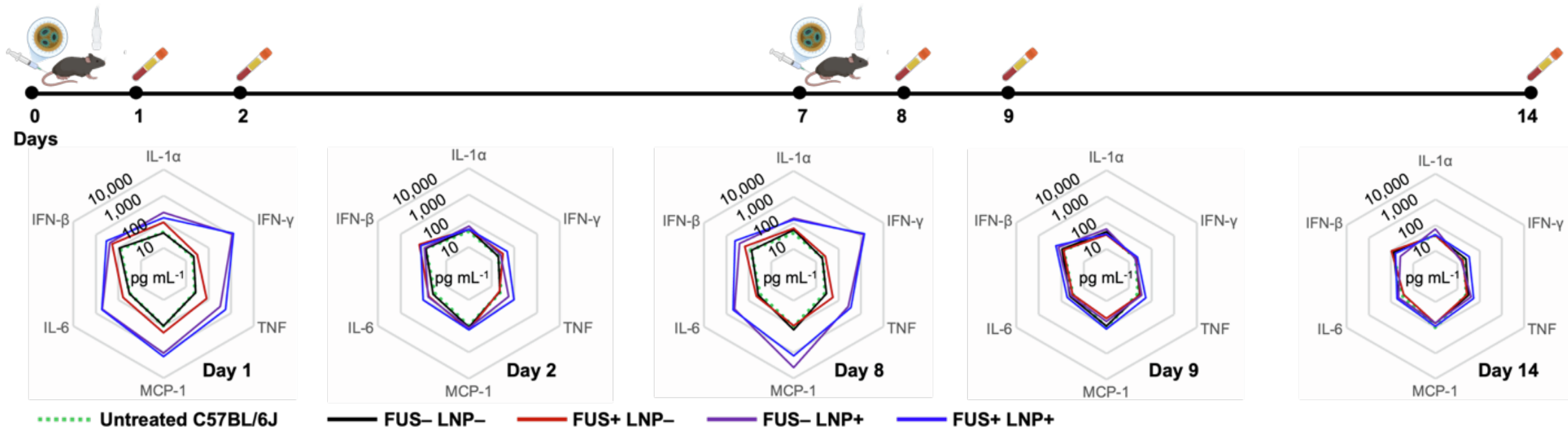

Figure S7. Plasma pro-inflammatory cytokine levels following treatment, with experimental timeline shown in the top panel (created with BioRender). "FUS-LNP-" indicates APOE4KI mice without any treatment, "FUS+LNP-" APOE4KI mice received FUS alone without i.v. LNPs, "FUS-LNP+" APOE4KI mice received i.v. LNPs alone without FUS, and "FUS+LNP+" APOE4KI mice received both FUS and i.v. LNPs. "Untreated C57BL/6J" denotes wild-type C57BL/6J mice without any treatment.

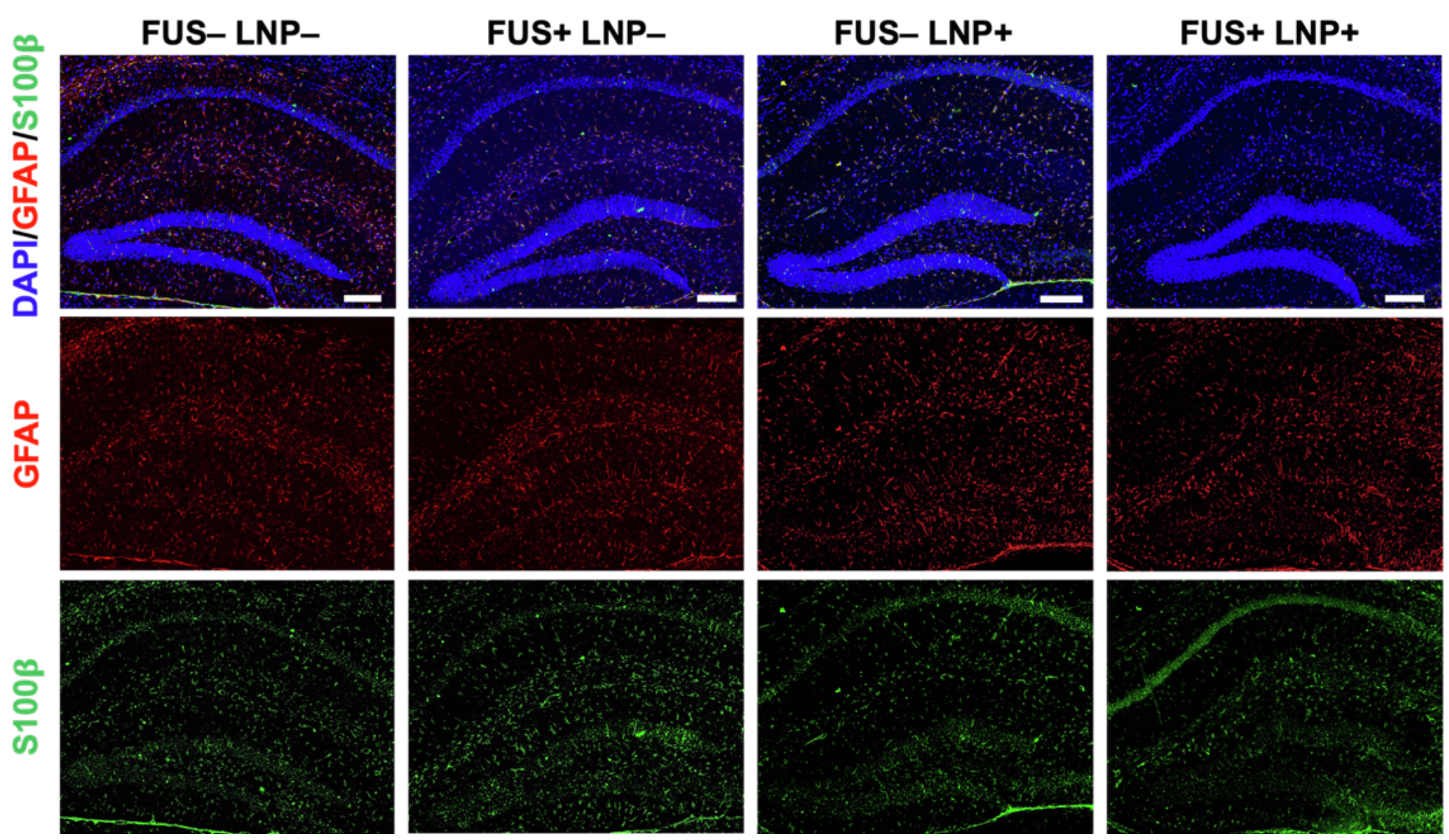

Figure S8. Representative confocal images of IF staining for GFAP (red), S100β (green), and DAPI (blue) in the hippocampus. Scale bars, 200 μm.

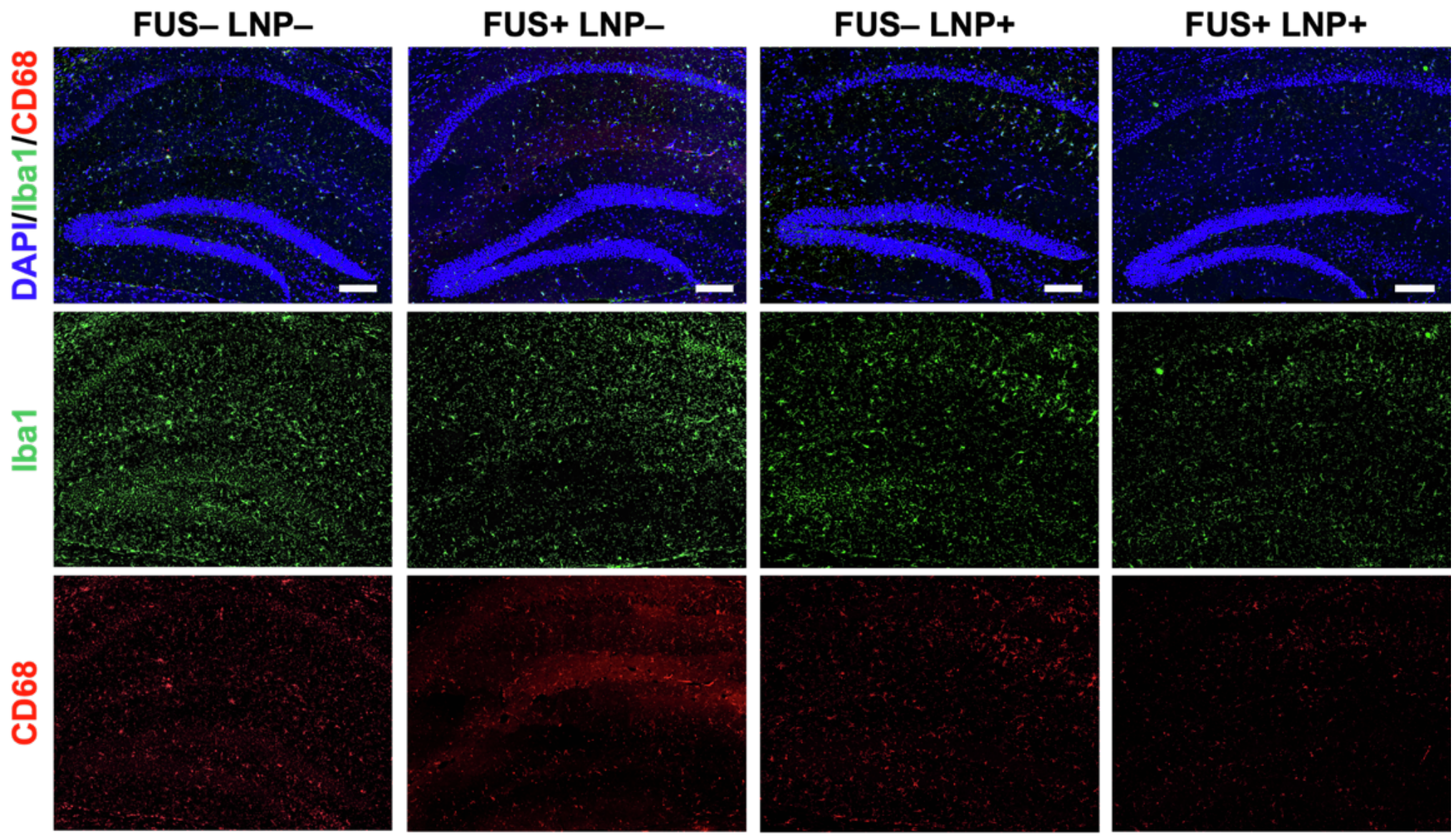

Figure S9. Representative confocal images of IF staining for Iba1 (green), CD68 (red), and DAPI (blue) in the hippocampus. Scale bars, 200 μm.

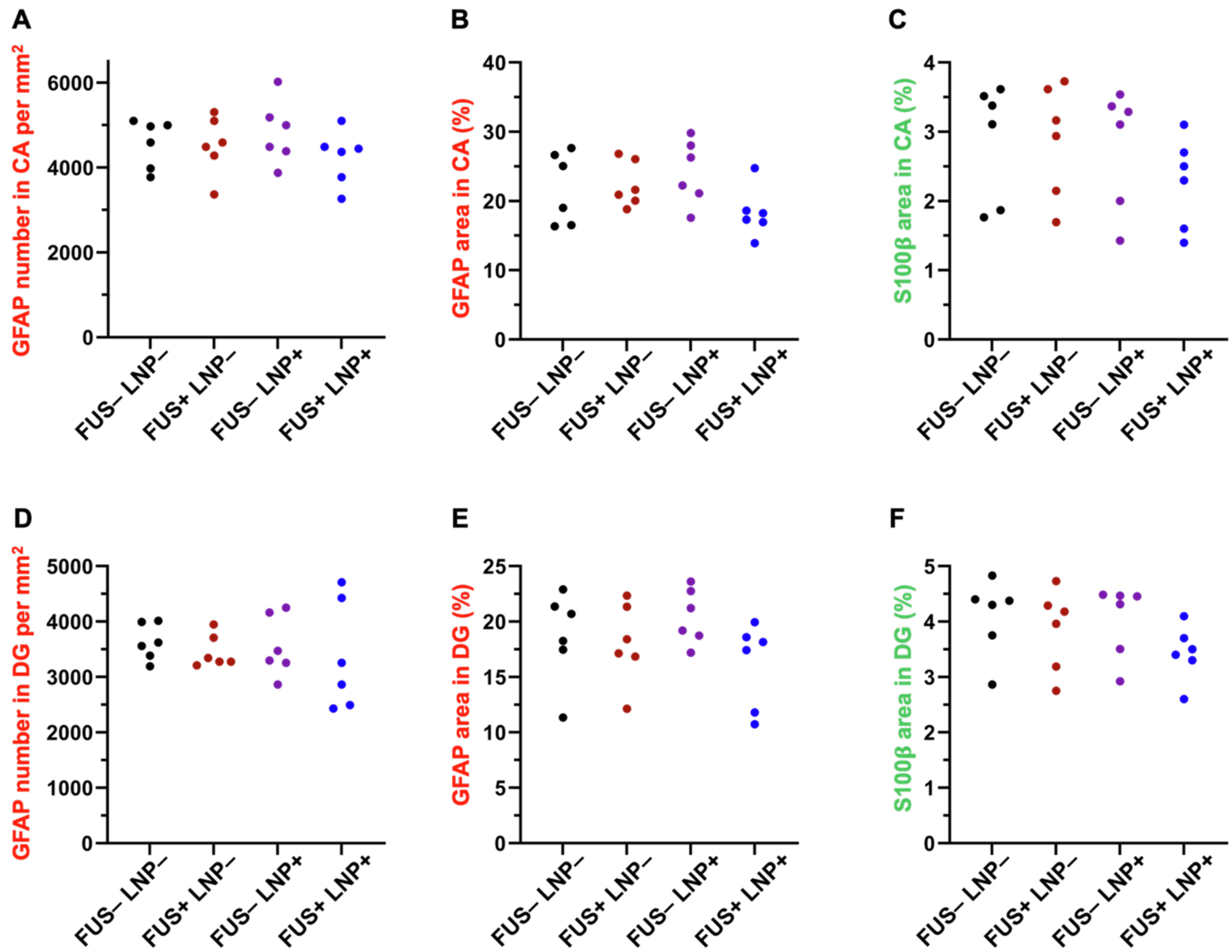

Figure S10. Raw values used to quantify astrocyte markers in the CA and DG regions. Raw values for the number of GFAP (A), coverage area of GFAP (B), and coverage area of S100β (C) in the CA region, and the number of GFAP (D), coverage area of GFAP (E), and coverage area of S100β (F) in the DG region.

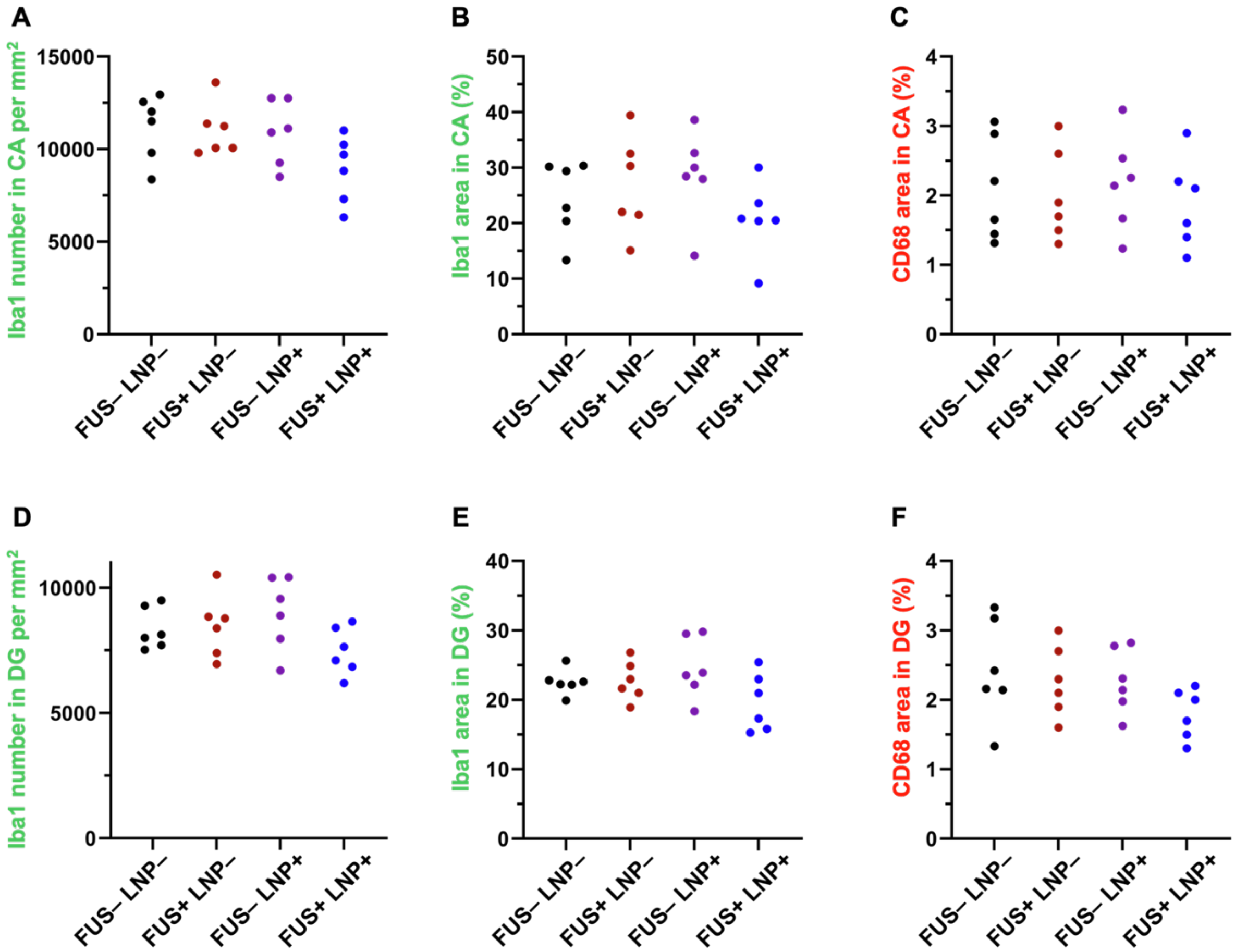

Figure S11. Raw values used to quantify microglia markers in the CA and DG regions. Raw values for the number of Iba1 (A), coverage area of Iba1 (B), and coverage area of CD68 (C) in the CA region, and the number of Iba1 (D), coverage area of Iba1 (E), and coverage area of CD68 (F) in the DG region.

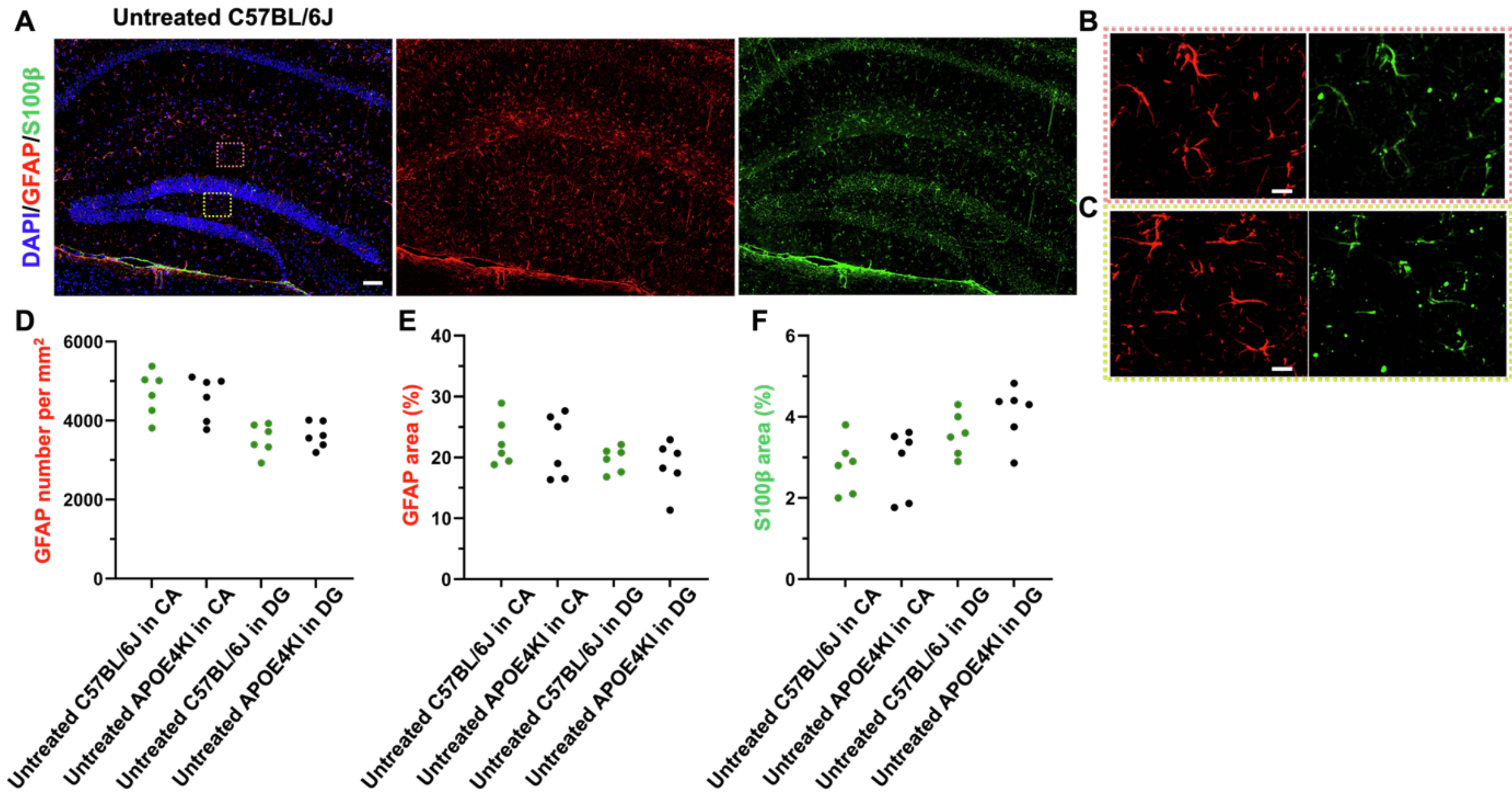

Figure S12. IF staining of astrocyte markers in untreated wild-type C57BL/6J mice. (A-C) IF staining of untreated wild-type C57BL/6J mice for GFAP (red), S100β (green), and DAPI (blue). (A) Representative confocal images in the hippocampus. Scale bars, 200 µm. Pink and yellow boxes indicate ROIs in the CA (B) and DG (C) regions, respectively. Scale bars, 20 µm. (D-F) Raw values for number of GFAP (D), coverage area of GFAP (E), and coverage area of S100β (F) in the CA and DG regions of untreated wild-type C57BL/6J mice and untreated APOE4KI mice (FUS-LNP-).

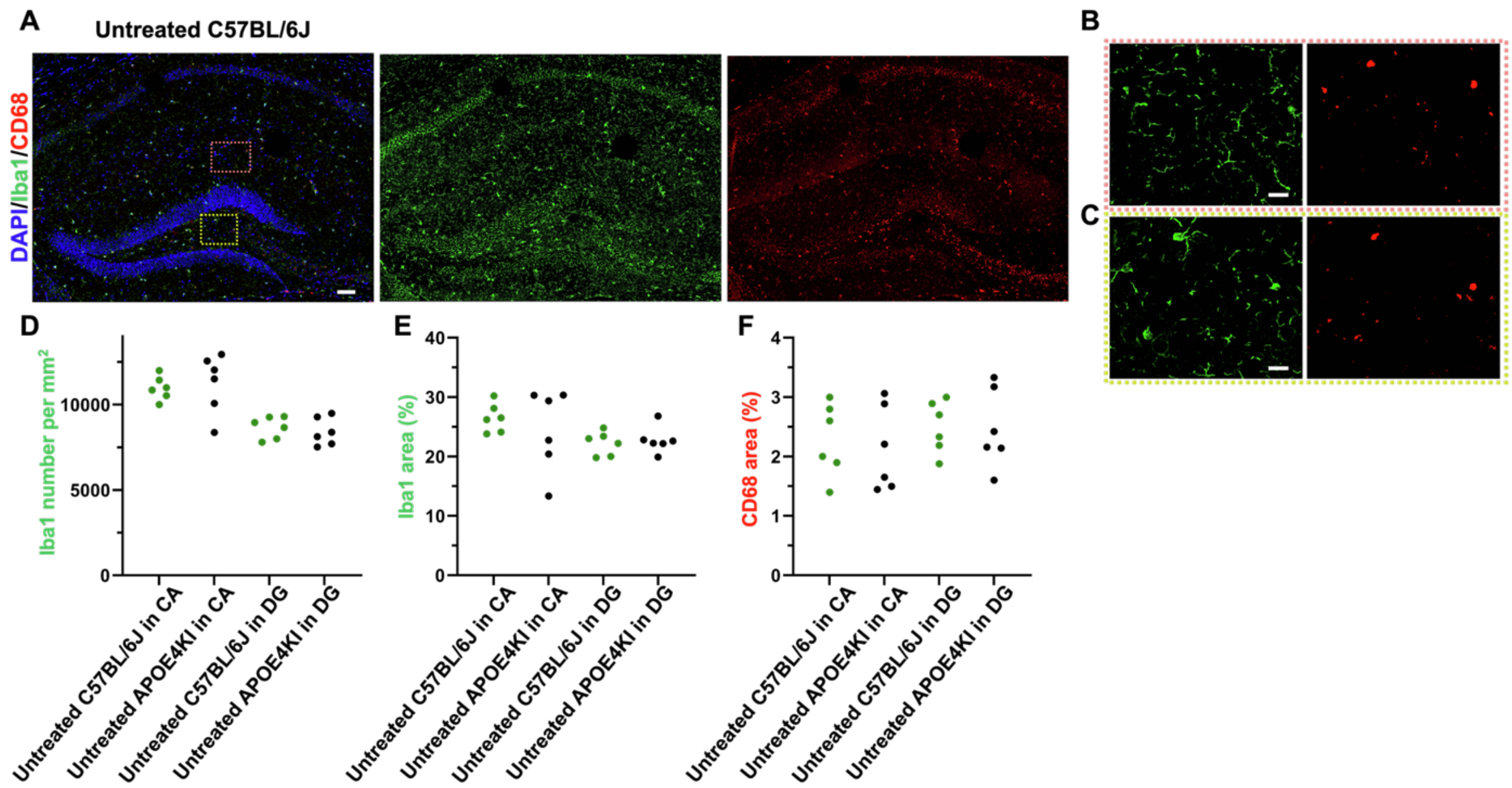

Figure S13. IF staining of microglia markers in untreated wild-type C57BL/6J mice. (A-C) IF staining of untreated wild-type C57BL/6J mice for Iba1 (green), CD68 (red), and DAPI (blue). (A) Representative confocal images in the hippocampus. Scale bars, 200 µm. Pink and yellow boxes indicate ROIs in the CA (B) and DG (C) regions, respectively. Scale bars, 20 µm. (D-F) Raw values for number of Iba1 (D), coverage area of Iba1 (E), and coverage area of CD68 (F) in the CA and DG regions of untreated wild-type C57BL/6J mice and untreated APOE4KI mice (FUS-LNP-).

SUPPLEMENTAL METHODS

Characterization of pDNA-LNPs

Particle size and zeta potential of pDNA-LNPs were measured using ZetaSizer Nano ZS90 (Malvern Panalytical), and values are reported as mean ± SD (n = 3). Encapsulation efficiency (EE) of pDNA in LNPs was quantified using Quant-iT™ PicoGreen™ dsDNA Assay Kits (Invitrogen). The concentration of unencapsulated pDNA was measured in intact pDNA-LNPs, whereas the concentration of total pDNA was measured after disruption of the LNPs with Triton X-100. EE was calculated according to the following formula:

$$\mathrm{EE}\ (\%) = \frac{\text{total pDNA} - \text{unencapsulated pDNA}}{\text{total pDNA}} \times 100 \qquad \text{[S1]}$$

Mice

All animal studies were performed in accordance with protocols AC-AABU2665, AC-AABG4559, and AC-AABX4654 approved by the Columbia University Institutional Animal Care and Use Committee (IACUC). Transgenic APOE4KI mice (JAX Stock No. 027894), which carry homozygous humanized APOE4 alleles [B6(SJL)-Apoe$^{tm1.1(APOE*4)Adiuj}$/J], were purchased from The Jackson Laboratory. The wild-type C57BL/6J mice (JAX Stock No. 000664) were used as untreated controls. All animals were housed with littermates in a temperature-controlled facility under a 12-hour light/dark cycle and were provided ad libitum access to standard chow and water.

In vivo gene editing validation

Genomic DNA and RNA were extracted from the left and right hippocampi using the Quick DNA/RNA Miniprep Plus Kit (Zymo) according to the manufacturer's instructions.

Genomic DNA was amplified using Platinum SuperFi II PCR Master Mix (Invitrogen) with the following cycling conditions: 98 °C for 10 s, 60 °C for 10 s, and 72 °C for 15 s, repeated for 35 cycles. PCR amplicons were purified using the GeneJET Gel Extraction and DNA Cleanup Micro Kit (Thermo Scientific) and quantified with Quant-iT™ PicoGreen™ dsDNA Assay Kits (Invitrogen) according to the manufacturer's protocols. Purified amplicons were sequenced by Plasmidsaurus, and the sequencing data were analyzed using CRISPResso2. Editing efficiency was calculated from amplicon deep sequencing as the percentage of sequencing reads containing insertions, deletions, and substitutions around each predicted cut site.

Extracted RNA was reverse-transcribed into cDNA using the iScript cDNA Synthesis Kit (Bio-Rad) according to the manufacturer's instructions. Reverse transcription-quantitative polymerase chain reaction (RT-qPCR) was performed on QuantStudio 3 Real-Time PCR System (Applied Biosystems) using SsoAdvanced SYBR Green Supermix (Bio-Rad) according to the manufacturer's protocol. APOE4 mRNA expression was normalized to the housekeeping gene 18S rRNA. For each mouse, the relative ratio was calculated by comparing the normalized APOE4 expression level in the left hippocampus (FUS+LNP+) with that in the right hippocampus (FUS-LNP+) to compare between the sonicated and contralateral hippocampus within the same animal, in order to account for inter-animal variability. In the control group, the ratio was calculated by comparing the left hippocampus (FUS+LNP-) with the right hippocampus (FUS-LNP-).

RNA *in situ* hybridization (ISH)

RNA ISH was performed on paraffin-embedded brain sections. Sections were deparaffinized and pretreated using the RNAscope Multiplex Fluorescent Reagent Kit v2 (Advanced Cell Diagnostics, 323100) according to the manufacturer's instructions. Probes targeting Mm-Gfap (313211),

saCas9 (501621-C2), and Hs-APOE (433091-C3) were used in combination with TSA Vivid Fluorophore 520 (323271), TSA Vivid Fluorophore 570 (323272), and TSA Vivid Fluorophore 650 (323273) according to the manufacturer's protocol.

To quantify *SaCas9* mRNA expression across different cell types, including astrocytes, microglia, neurons, oligodendrocytes, and endothelial cells, ISH was combined with immunofluorescence according to the manufacturer's protocol. The following primary antibodies were used: chicken anti-GFAP (1:500, Abcam, ab4674), rabbit anti-Iba1 (1:500, Wako, 019-19741), rabbit anti-NeuN (1:800, Abcam, ab177487), rabbit anti-Olig2 (1:200, Abcam, ab109186), and rabbit anti-CD31 (1:1000, Abcam, ab281583). The following secondary antibodies were used: Alexa Fluor 488 goat anti-rabbit (1:500, Abcam, ab150077) and Alexa Fluor 594 goat anti-chicken (1:500, Abcam, ab150172). As for *SaCas9* mRNA, the saCas9 (501621-C2) probe was used in combination with TSA Vivid Fluorophore 650 (323273).

Imaging was performed using a Nikon Ti2 inverted microscope equipped with an AXR resonant spectral scanning confocal unit at 60× magnification. Cells were manually counted and classified as positive for a target gene if they contained at least five RNA spots within the corresponding cell-marker-defined region.

Enzyme-linked immunosorbent assay (ELISA)

ApoE4 protein levels in brain lysates from the left and right hippocampi were quantified using the Human Apolipoprotein E4 ELISA Kit (Abcam) according to the manufacturer's instructions. ApoE4 expression was normalized to the housekeeping protein GAPDH using the Mouse GAPDH ELISA Kit (AFG Scientific) according to the manufacturer's protocol. For each mouse, the relative ratio was calculated by comparing the normalized apoE4 expression level in the left hippocampus

(FUS+LNP+) with that in the right hippocampus (FUS-LNP+). In the control group, the ratio was calculated by comparing the left hippocampus (FUS+LNP-) with the right hippocampus (FUS-LNP-).

Immunofluorescence (IF) staining

Collected brains were post-fixed in 4% PFA, embedded in paraffin, and coronally sectioned at a thickness of 5 μm. Sections were deparaffinized in xylene, rehydrated through a graded ethanol series, and subjected to antigen retrieval using eBioscience IHC Antigen Retrieval Solution (Invitrogen). Sections were then rinsed in TBS-Tween 20 (TBST, Thermo Scientific) and blocked with 10% goat serum in TBST for 1 h at room temperature. Primary antibodies diluted in blocking buffer were applied to the sections, which were then incubated overnight at 4 °C. The following primary antibodies were used: rabbit anti-ApoE (1:500, Abcam, ab52607), chicken anti-GFAP (1:500, Abcam, ab4674), rabbit anti-S100β (1:500, Abcam, ab52642), rabbit anti-Iba1 (1:500, Wako, 019-19741), and chicken anti-CD68 (1:500, Abcam, ab318303). After three 10-min washes in TBST, sections were incubated with secondary antibodies diluted in blocking buffer for 2 h at room temperature. The following secondary antibodies were used: Alexa Fluor 488 goat anti-rabbit (1:500, Abcam, ab150077) and Alexa Fluor 594 goat anti-chicken (1:500, Abcam, ab150172). Finally, sections were washed three times for 10 min each in TBST and mounted with ProLong Gold Antifade Mountant with DAPI (Invitrogen).

Imaging was performed using a Nikon Ti2 inverted microscope equipped with an AXR resonant spectral scanning confocal unit at 60× magnification. Colocalization of ApoE4 and GFAP, as well as the number and coverage area of GFAP-positive and Iba1-positive cells, were quantified using Imaris 10.2 (Oxford Instruments) with the AI-based Filament Tracer for linear branching

networks. The coverage areas of S100β and CD68 were quantified using ImageJ. Quantification was performed separately for the cornu ammonis (CA) and dentate gyrus (DG) regions of the hippocampus, and marker abundance was expressed as cell number per mm^2 or coverage area (%). For each mouse, the relative ratio of each marker in the left hippocampus (FUS+LNP+) to that in the right hippocampus (FUS-LNP+) was calculated. In the control group, the ratio of the left hippocampus (FUS+LNP-) to the right hippocampus (FUS-LNP-) was calculated.

Pro-inflammatory cytokine measurements

Blood was collected from the facial vein using a sterile 5 mm lancet into capillary blood collection tubes pre-coated with lithium heparin at designated time points after treatment. Samples were centrifuged at 2,000 × g for 10 min at 4 °C, and the plasma supernatant was collected and stored at -80 °C until analysis. Plasma cytokine levels were quantified by ELISA according to the manufacturers' instructions using kits from Invitrogen for IFN-β, IL-1α, IFN-γ, TNF-α, MCP-1, and IL-6.

Statistics

Statistical analyses were performed using Prism 9.0 (GraphPad). Data are presented as mean ± standard error of the mean (s.e.m.). Statistical significance was assessed using unpaired two-tailed Student's t-tests or two-way ANOVA followed by Dunnett's multiple comparisons test comparing each group with the FUS-LNP- control group. Differences were considered statistically significant at $*P < 0.05$, $**P < 0.01$, $***P < 0.001$, and $****P < 0.0001$.
